# Plant microbiomes harbor potential to promote nutrient turnover in impoverished substrates of a Brazilian biodiversity hotspot

**DOI:** 10.1038/s41396-022-01345-1

**Published:** 2022-12-20

**Authors:** Antonio P. Camargo, Rafael S. C. de Souza, Juliana Jose, Isabel R. Gerhardt, Ricardo A. Dante, Supratim Mukherjee, Marcel Huntemann, Nikos C. Kyrpides, Marcelo F. Carazzolle, Paulo Arruda

**Affiliations:** 1 Centro de Biologia Molecular e Engenharia Genética, Universidade Estadual de Campinas (UNICAMP), 13083-875 Campinas, SP Brazil; 2 Departamento de Genética e Evolução, Instituto de Biologia, Universidade Estadual de Campinas (UNICAMP), 13083-970 Campinas, SP Brazil; 3 Genomics for Climate Change Research Center (GCCRC), Universidade Estadual de Campinas (UNICAMP), 13083-875 Campinas, SP Brazil; 4 US Department of Energy Joint Genome Institute, Lawrence Berkeley National Laboratory, Berkeley, CA USA; 5 Symbiomics Microbiome Solutions, Florianópolis, SC 88050-000 Brazil; 6 Embrapa Agricultura Digital, 13083-886 Campinas, SP Brazil

**Keywords:** Metagenomics, Soil microbiology

## Abstract

The substrates of the Brazilian *campos rupestre*s, a grassland ecosystem, have extremely low concentrations of phosphorus and nitrogen, imposing restrictions to plant growth. Despite that, this ecosystem harbors almost 15% of the Brazilian plant diversity, raising the question of how plants acquire nutrients in such a harsh environment. Here, we set out to uncover the taxonomic profile, the compositional and functional differences and similarities, and the nutrient turnover potential of microbial communities associated with two plant species of the *campos rupestres*-dominant family *Velloziaceae* that grow over distinct substrates (soil and rock). Using amplicon sequencing data, we show that, despite the pronounced composition differentiation, the plant-associated soil and rock communities share a core of highly efficient colonizers that tend to be highly abundant and is enriched in 21 bacterial families. Functional investigation of metagenomes and 522 metagenome-assembled genomes revealed that the microorganisms found associated to plant roots are enriched in genes involved in organic compound intake, and phosphorus and nitrogen turnover. We show that potential for phosphorus transport, mineralization, and solubilization are mostly found within bacterial families of the shared microbiome, such as *Xanthobacteraceae* and *Bryobacteraceae*. We also detected the full repertoire of nitrogen cycle-related genes and discovered a lineage of *Isosphaeraceae* that acquired nitrogen-fixing potential via horizontal gene transfer and might be also involved in nitrification via a metabolic handoff association with *Binataceae*. We highlight that plant-associated microbial populations in the *campos rupestre*s harbor a genetic repertoire with potential to increase nutrient availability and that the microbiomes of biodiversity hotspots can reveal novel mechanisms of nutrient turnover.

## Background

**Fig. 1:**
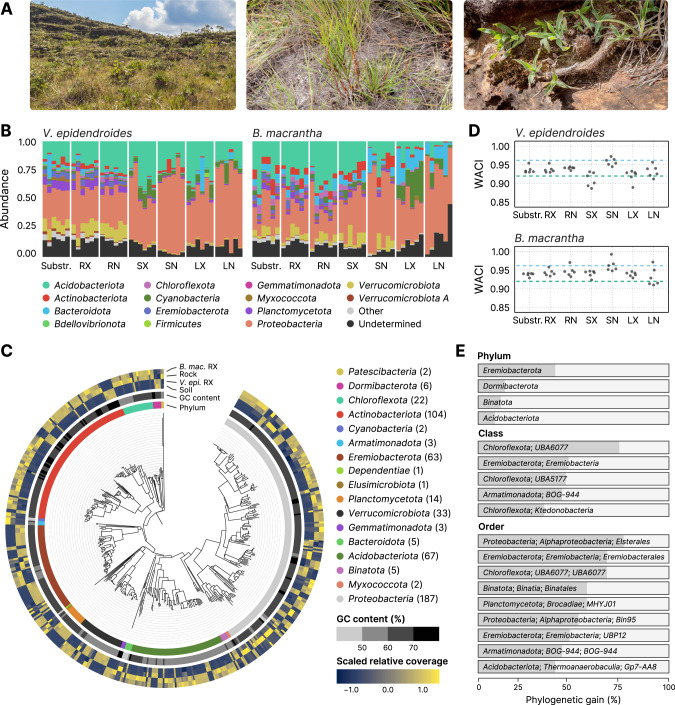
Composition and novelty of the *V. epidendroides* and *B. macrantha* microbiomes. **A** Sampling was conducted in the *campos rupestres* grasslands ecoregion (left). *Vellozia epidendroides* (center) specimens were collected in patches of shallow soil. *Barbacenia macrantha* (right) was found in a rocky area, where it grows over exposed rocks. **B** Community composition inferred from 16S rRNA gene ASVs at the phylum level. Samples were grouped according to their environment. Bar heights are proportional to the relative abundance of the phylum. Low abundance phyla (relative abundance < 2%) were grouped under the “Other” category. **C** Maximum-likelihood phylogenetic tree of the bacterial MAGs presented in this study, rooted at the *Patescibacteria* clade. The innermost ring indicates the phylum associated with each node. The center ring shows the genomic GC content. The outermost ring represents the scaled means of log-transformed relative genomic coverages across the four environments. **D** Weighted average community identity (WACI) computed from 16S rRNA gene ASV data. The blue and green dashed lines represent the median intra-rank 16S rRNA gene identity at the genus and family levels, respectively. **E** Phylogenetic gain (PG) contributed by the MAGs to different taxa at the phylum, class, and order levels. Only taxa with PG higher than the following cut-offs are shown: 5% at the phylum level, 30% at the class level, and 40% at the order level. RX root (external), RN root (internal), SX stem (external), SN stem (internal), LX leaf (external), LN leaf (internal).

The Brazilian *campos rupestres* constitute a grassland ecosystem located on the geologically old rocky outcrops of the central and eastern regions of Brazil (Fig. 1A, left). *Campos rupestres* soils are shallow, acidic, and severely nutrient-impoverished. Phosphorus (P), the major nutritional constraint in this environment, imposes a high acquisition cost to resident plants [1] as it is found in low concentrations, due to extreme weathering and leaching of the parent rock [2], and binding to iron and aluminum because of the low pH. Despite this abiotic constraint, the *campos rupestres* constitute a biodiversity hotspot with an average species density among the world’s highest, harboring thousands of endemic vascular plant species from highly specialized and phylogenetically clustered lineages [3, 4].

Plant adaptations to the nutritional scarcity of the *campos rupestres* substrates have been extensively studied. Members of the highly successful *Velloziaceae* family have been shown to use multiple strategies to cope with nutrient limitation, such as the formation of durable and well-defended structures [5], the efficient remobilization of P from senescent leaves, and the development of specialized radicular systems that enhance nutrient uptake via the secretion of carboxylates [6–8]. As a result of the plant-centric approach that has dominated the study of the *campos rupestres* nutrient dynamics, it remains unclear how plant growth is influenced by interactions with microorganisms.

Plant microbiomes play fundamental roles in shaping the host responses to biotic and abiotic stresses and modulating plant phenotypic plasticity. These microorganisms can form complex and stable associations that can determine plant speciation, geographic distribution patterns, and diversity to a much stronger degree than previously acknowledged [9]. This scenario evidences that plants cannot be perceived as isolated entities but as a unit formed by the host and its associated microbiota [10]. Such tight interactions indicate that these microbial communities are not random assemblages of microorganisms, and that intricate inter-species relationships shape the microbiome composition [11].

Microbiome-driven processes involved in nutrient acquisition are thought to have a decisive influence in naturally stressful environments, where plants are inclined to be more dependent on microbial communities for nutrient uptake than in nutrient-rich habitats, where resources are readily available [12]. In grasslands and savannahs, for example, nitrogen-fixing bacteria and mycorrhizal association contribute up to 20% of total nitrogen acquired by the vegetation [13]. In boreal forests, where nutrient availability is severely affected by low temperatures and soil pH, microbiomes were estimated to be responsible for most of the nitrogen and phosphorus acquired by plants [14, 15].

Previous reports showing that most *campos rupestres* plant species growing under P-limited substrates do not associate with mycorrhizal fungi [16] have strengthened the belief that these species rely solely on their own adaptations to acquire phosphorus. However, a high-throughput assessment of the diversity and functions of plant-associated microorganisms has never been conducted in the *campos rupestres*. Thus, an understanding of how microbial communities influence acquisition and turnover of nutrients in this environment remains unresolved.

Here, we investigated microbial communities associated with two species of *Velloziaceae* growing in distinct substrates that were previously shown to be nutrient-impoverished [17]: *Vellozia epidendroides* Mart. ex Schult. & Schult. f. and *Barbacenia macrantha* Lem, which are found growing in shallow soil patches (Fig. 1A, center) and over rocks (Fig. 1A, right), respectively. We hypothesized that these species select and form associations with microorganisms that promote growth via nutrient turnover and that the compositions of these beneficial communities depend on substrate type. Therefore, we inquired: (1) what is the composition and the novelty of the communities associated with *V. epidendroides* and *B. macrantha*; (2) what are the differences and similarities between the microbiomes associated with these species; (3) whether there is enrichment of genes linked to microbiota recruitment and nutrient turnover in the microbiomes of the roots; and (4) what are the mechanisms of phosphorus and nitrogen turnover encoded by the microbiomes of each of these species, how do they compare, and how is the shared microbiota involved.

Using sequencing data of the communities associated with the substrates and the plants, we show that these *campos rupestres Velloziaceae* are associated with novel and diverse microorganisms, many of which are shared between the two plant species. Our results also indicate that microbial functions linked to phosphorus and nitrogen turnover are present and enriched in the plant root-associated microorganisms. These findings are compatible with a model of active selection of beneficial microbial communities by plant hosts and provide new insights regarding mechanisms of plant-microbiome association in nutrient-depleted environments.

## Results

### The taxonomic landscape of *Velloziaceae* microbiomes was assessed through amplicon and MAG data

To profile the composition of microbial communities associated with *V. epidendroides* and *B. macrantha* we employed amplicon sequencing data of the 16S rRNA gene (for prokaryotes) and the internal transcribed spacer 2 (ITS2) region (for fungi). From substrate (soil and rock) and plant organ (external and internal compartments of the root, stem, and leaves) samples obtained from six individuals of each species [17] we identified 29,008 16S rRNA gene and 9,153 ITS unique amplicon sequence variants (ASVs), which were assigned to three archaeal, 38 bacterial (Fig. 1B), and 13 fungal (Supplementary Fig. 1A) phyla. These data revealed that, at high ranks, the taxonomic profiles of the *campos rupestres* microbiomes echoed that of global soil surveys for bacteria, archaea, and fungi [18–20], with communities dominated by phyla such as *Proteobacteria*, *Acidobacteriota*, *Verrucomicrobiota*, *Ascomycota* and *Basidiomycota*.

In addition to the amplicon datasets, four metagenomes (three samples and a co-assembly) were assembled for each environment (soil-, rock-, and external root-associated samples), totaling 16 metagenomes with 25.8 Gbp (Supplementary Table 1). From these assemblies, 522 metagenome-assembled genomes (MAGs) of microorganisms that dwell in substrates and root surfaces were recovered and assigned to one archaeal and 17 bacterial phyla (Fig. 1C, Supplementary Table 1). Further clustering based on average nucleotide identity (ANI) [21] revealed 331 species-level clusters (≥ 95% ANI). The metagenomes recovered 16.3% to 42.4% of the total sequence diversity across different samples, while MAGs recovered from 10.8% to 39.1%. Additionally, most unassembled sequences were from populations closely related to those present in the metagenomes; sequence complexity was the main factor hindering metagenomic assembly (Supplementary Note 1, Supplementary Table 3).

### Taxonomic novelty of *campos rupestres* microbiomes

As the *campos rupestres* possess a high degree of endemism, we set out to estimate the taxonomic novelty of the investigated communities by comparing the sequences of the retrieved ASVs to public taxonomy databases (GTDB for prokaryotes and UNITE for fungi). Taxonomy could not be assigned at the family level to 46.5% of 16S rRNA gene and 77.5% of ITS ASVs and at the phylum level to 25.8% of 16S rRNA gene and 48.7% of ITS ASVs (Fig. 1B, Supplementary Fig. 1A). To quantify the community-level taxonomic novelty we devised the weighted average community identity (WACI) metric, which represents the abundance-weighted average identity of alignments between ASVs and their best matches in a reference database. Bacterial communities exhibited WACI around 91.8% to 95.8% (10th and 90th percentiles, Fig. 1D), suggesting that they are dominated by novel genera and families (median within-rank identity of 96.4% and 92.3%, respectively [22]). There were no significant differences between the WACI of below-ground (substrate and root) and above-ground (stem and leaf) bacterial communities. However, below-ground fungal communities were significantly more novel in both plants (Supplementary Fig. 1B, linear mixed-effects model [LMM] *p* value < 0.05; *V. epidendroides* ω^2^ ≈ 0.64; *B. macrantha* ω^2^ ≈ 0.55) and had lower WACI (median: 87.3% in *V. epidendroides* and 90.8% in *B. macrantha*).

Novelty within MAGs was quantified using the phylogenetic novelty metric, which represents the phylogenetic gain (PG; defined as the total branch length added by a set of genomes to a clade) brought by the MAGs to their lineages (Fig. 1E). Out of the 522 genomes, 268 (51.3%) belonged to novel genera, 66 (12.6%) belonged to new families, and 19 (3.6%) were assigned to new orders. These data contributed a substantial amount of phylogenetic novelty to known taxa (Supplementary Table 4), including some understudied phyla such as the *Eremiobacterota*, *Dormibacterota*, and *Binatota*, that were significantly expanded (PG: 40.4%, 13.9%, and 11.7%). A substantial amount of phylogenetic diversity was added to *Acidobacteriota* (PG: 8.6%), a phylum consistently found among abundant taxa in soils across the world [18–20]. We also found that the *Elsterales*, an order within the *Proteobacteria* that was previously found to be highly enriched in P-limited soils [23], was greatly expanded (PG: 76.3%).

### Comparison of the *V. epidendroides* and *B. macrantha*-associated communities

Because *V. epidendroides* and *B. macrantha* grow over contrasting substrates, we set out to compare their microbiomes in terms of diversity and taxonomic composition. Assessment of 16S rRNA gene ASV data of plant-associated microbiomes revealed that the average alpha diversity (Pielou’s equitability index [24] and richness) did not significantly differ between the prokaryotic communities associated with the two plants when discounting the effects of each tissue (Supplementary Fig. 2A, B). Conversely, *V. epidendroides*-associated fungal communities exhibited significantly higher alpha diversity according to both estimates (LMM *p* value < 0.05; richness *ω*^2^ ≈ 0.39; equitability *ω*^2^ ≈ 0.3).

**Fig. 2:**
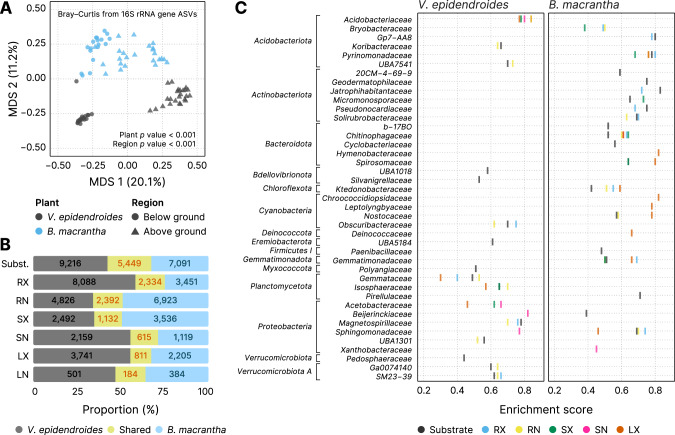
The differentiation between *V. epidendroides* and *B. macrantha* microbiomes is taxonomically structured. **A** Multidimensional scaling of the Bray–Curtis dissimilarities computed from 16S rRNA gene ASV abundance data. Samples are colored according to their associated plant species, and shape indicates whether they were from below ground (substrate and root) or above ground (stem and leaves) environments. The *p* values of the groupings were obtained from PERMANOVA tests. **B** Bar plots representing the fraction of *V. epidendroides*-exclusive, shared, and *B. macrantha*-exclusive 16S rRNA gene ASVs across all sample types. The absolute numbers of ASVs within each group are shown. **C** Enrichment of bacterial families (grouped by phyla) in one or the other plant across all environments (circle colors). The enrichment score in the x-axis was computed using the Kolmogorov–Smirnov test and represent the deviation from a null model where ASVs from a given family are uniformly distributed in a list ranked by the ratio between the abundances in each plant. No family was found to be enriched in the internal leaf communities of either plant. RX root (external), RN root (internal), SX stem (external), SN stem (internal), LX leaf (external), LN leaf (internal).

Comparisons of community composition, performed using beta diversity metrics, showed that the microbiota associated with *V. epidendroides* and *B. macrantha* significantly differed (Fig. 2A, Supplementary Fig. 2C, D; PERMANOVA *p* value < 0.001). Indeed, 74.9–88.0% of the 16S rRNA gene AVSs and 74.7–95.8% of the ITS ASVs were exclusive to one or the other plant across all sample types (Fig. 2B, Supplementary Fig. 2E). The differences in 16S rRNA gene ASV abundances between the two plant-associated communities revealed that 41 families from 15 phyla were significantly enriched in the microbiomes of one of the plants (Fig. 2C), indicating that the differentiation was taxonomically structured. Six different *Actinobacteriota* families were exclusively enriched in *B. macrantha*. This structured differentiation was also verified in the MAGs, as phylogenetic proximity was significantly correlated with abundance profile similarity (Mantel test *p* value < 0.001, Fig. 1C).

**Fig. 3:**
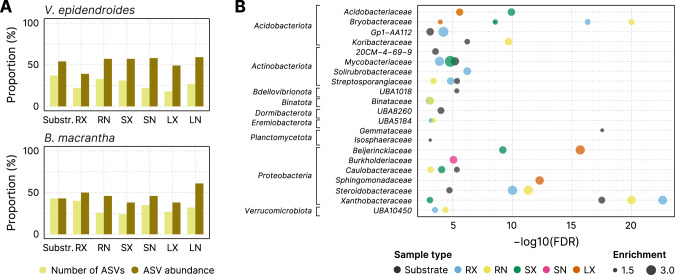
*V. epidendroides* and *B. macrantha* share a core microbiome that encompasses multiple families of efficient colonizers. **A** Proportion of the total number of 16S rRNA gene ASVs (light yellow) and of the ASV abundance (dark yellow) shared between the communities associated with both plants. **B** Bacterial families (grouped by phyla) enriched within the shared ASV sets. The x-axis shows the false discovery rate (FDR) obtained from hypergeometric tests. The extent of the enrichment for each family, represented by the circle areas, was quantified as the ratio between the number of ASVs in the shared fraction and the number of ASVs observed in both the shared and exclusive fractions. No family was found to be enriched in the internal leaf communities of either plant. RX root (external), RN root (internal), SX stem (external), SN stem (internal), LX leaf (external), LN leaf (internal).

Considering that both plant species cope with multiple environmental stresses, we hypothesized that they might share a common set of microorganisms that are adapted to the *campos rupestres* harsh environment. We found that, despite the extensive compositional differentiation, *V. epidendroides* and *B. macrantha* shared a fraction of both 16S rRNA gene (12–25%, Fig. 3A) and ITS (4.2–25.3%, Supplementary Fig. 2F) ASVs. The shared microorganisms tended to have higher average abundances across most tissues, often comprising more than half of the total community abundance. Additionally, 21 bacterial families from 9 phyla were enriched within the shared 16S rRNA gene ASV sets (Fig. 3B).

### Evaluation of microbiota recruitment capability by hosts and the microbial carbon cycling

**Fig. 4:**
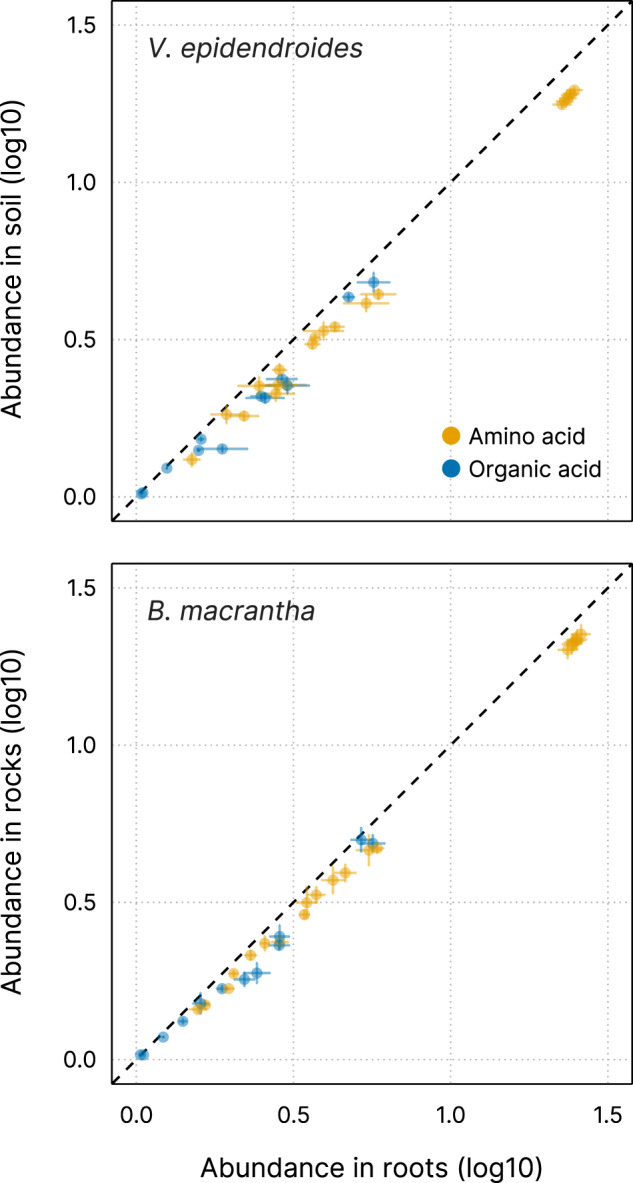
Mean total abundance (sum of RPKGs) of the investigated transporter genes in the roots (x-axis) and substrates (y-axis) of both plants. Circles are colored according to their assigned substrate class: amino acids (23 substrates) and organic acids (16 substrates). Horizontal and vertical lines represent the standard error of the mean in roots and substrates, respectively.

Plants recruit beneficial microorganisms dynamically using root exudates rich in organic compounds such as amino acids and organic acids [20, 25, 26], which positively select microbes that are able to take in these molecules. To investigate whether the microbial communities associated with *V. epidendroides* and *B. macrantha* have the potential to be actively recruited through this mechanism, the abundances of genes encoding organic substrate transporters (Supplementary Table 5) were estimated across all substrate and rhizosphere metagenomes. We found that the abundances of the evaluated transporters were systematically higher in the rhizosphere communities (Fig. 4, LMM *p* value < 0.001; *ω*^2^ ≈ 0.14). Transporter enrichment in the rhizosphere occurred for all organic substrates that were evaluated (Supplementary Table 6). Although the microbial communities associated with the two plant species did not significantly differ in total transporter abundance, they exhibited differing taxonomic profiles (Supplementary Fig. 3A).

Because the soil substrate contains higher organic matter content than the exposed rocks [17], we investigated the carbon cycling potential of the *V. epidendroides* and *B. macrantha* microbiomes with respect to carbohydrate degradation and carbon dioxide fixation (Supplementary Note 2, Supplementary Fig. 3, Supplementary Table 7). We found that genes associated with carbohydrate turnover were more abundant in the *B. macrantha*-associated communities (Supplementary Fig. 3D). In addition, although the microbiomes of both species exhibited potential for autotrophy, we found that photosynthetic bacteria were much more abundant in the rock-dwelling communities (Supplementary Fig. 3E, F). The photosynthetic *Chroococcidiopsidaceae* family, for example, was found to be highly enriched in *B. macrantha*-associated communities (Fig. 2C) and was represented within the MAGs retrieved from rock metagenomes. Through metabolic potential inference we identified 38 autotrophic MAGs belonging to seven different phyla (Supplementary Table 8).

### Investigation of the phosphorus turnover potential by *Velloziaceae* microbiomes

**Fig. 5:**
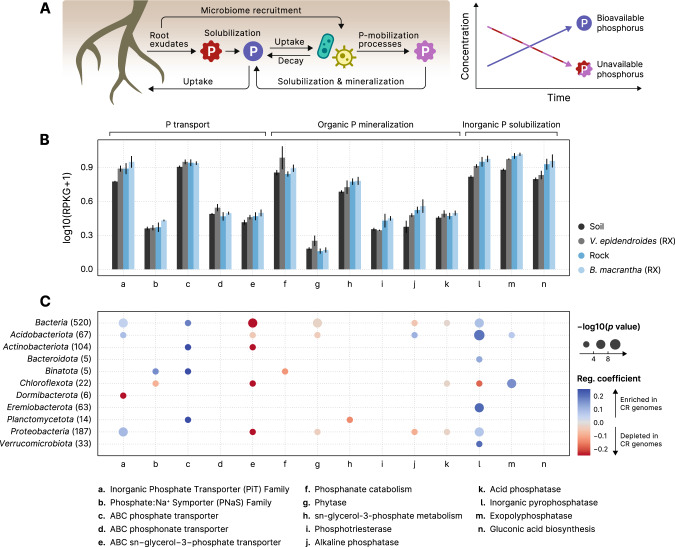
Root-associated microbiomes exhibit increased P-turnover potential in comparison to substrate communities. **A** Root exudates both solubilize phosphorus (P) in the plant substrate and recruit microorganisms that consume this nutrient. As the recruited microbes can mobilize phosphorus that would otherwise be unavailable for the plants (in pink), the total bioaccessible phosphorus concentration increases over time. **B** Mean total abundances (sum of RPKGs) of proteins and pathways involved in processes linked to phosphate turnover (transport, mineralization, and solubilization) in the substrate and root-associated communities. Abundances of multiprotein complexes (*pstABCS*, *phnCDE*, *ugpABCE*, and *phnGHIJKLM*) were computed by averaging the abundances of their subunits. Vertical lines represent the standard error of the mean. **C** Phylogenetic regressions of the number of phosphate turnover-related genes. The MAGs retrieved in this study were compared to GTDB genomes to identify differences in the numbers of gene copies associated with phosphorus turnover. The color scale indicates the magnitude of the enrichment (blue) or depletion (red) of each process in the MAGs, and the area of the circles represent the statistical significance of the regression coefficient. Regression coefficients with *p* value > 0.05 are omitted. Phylogenetic regressions were performed on the whole set of bacterial genomes and on the phyla containing at least 5 MAGs. RX root (external), CR *campos rupestres*.

Microorganisms encode diverse mechanisms for P-mobilization [27], and as their biomass turns over through time, P becomes available to plants [28] (Fig. 5A). To investigate the P-turnover potential of the *V. epidendroides* and *B. macrantha* microbiomes, we measured the total abundance of genes involved in environmental P mobilization (Supplementary Table 9). We found 14 processes that were represented in the metagenomes of both plants (Fig. 5B) and are encoded by diverse taxa (Supplementary Fig. 4A). Additionally, a systematic increase in the abundances of P turnover processes in the rhizospheres relative to their adjacent substrates was verified (Fig. 5B, LMM *p* value < 0.001; *ω*^2^ ≈ 0.12).

Despite extensive microbiome specialization in the two plant species, 12 out of 15 families with the highest total abundances of genes involved in P turnover were found to be significantly enriched in their shared associated microbiota fraction (Table 1, Fig. 3B). We also verified that different taxa might contribute to P mobilization using distinct mechanisms. For example, the *Bryobacteraceae* exhibited a high abundance of the *gcd* gene, involved in the synthesis of gluconic acid. This molecule that solubilizes P by chelating cations bound to recalcitrant phosphate [27, 29]. In contrast, the *UBA5184* had high abundances of genes encoding exopolyphosphatase [30] and inorganic pyrophosphatase [31], enzymes that release P from inorganic phosphate polymers. Among the families with the highest P turnover potential, 60% (9 out of 15) had PG higher than 10%.

**Table 1:** The top 15 families with the highest average total abundance of processes associated with P turnover.

Family	Phylum	*V. epidendroides*		*B. macrantha*		All samples	Family PG (%)
		Soil	RX	Rock	RX		
Undetermined	Undetermined	27.52	32.25	27.39	28.79	28.99	N/A
***Xanthobacteraceae***	***Proteobacteria***	**8.15**	**11.40**	**13.84**	**15.39**	**12.20**	**16.23**
***Bryobacteraceae***	***Acidobacteriota***	**2.54**	**3.03**	**6.30**	**7.83**	**4.92**	**32.15**
***UBA5184***	***Eremiobacterota***	**5.92**	**6.31**	**3.64**	**3.23**	**4.77**	**47.05**
*Acetobacteraceae*	*Proteobacteria*	3.53	4.50	3.12	6.30	4.36	18.00
***Solirubrobacteraceae***	***Actinobacteriota***	**0.83**	**2.01**	**6.60**	**6.39**	**3.96**	**34.32**
***Burkholderiaceae***	***Proteobacteria***	**3.77**	**7.47**	**1.71**	**2.00**	**3.74**	**2.01**
***Beijerinckiaceae***	***Proteobacteria***	**2.93**	**3.42**	**3.36**	**4.20**	**3.48**	**13.14**
*Reyranellaceae*	*Proteobacteria*	2.60	2.28	1.45	0.88	1.80	34.50
***Streptosporangiaceae***	***Actinobacteriota***	**1.23**	**1.12**	**1.94**	**1.84**	**1.53**	**6.43**
***Steroidobacteraceae***	***Proteobacteria***	**1.99**	**1.66**	**1.14**	**1.28**	**1.52**	**24.15**
***Acidobacteriaceae***	***Acidobacteriota***	**2.23**	**2.18**	**0.71**	**0.79**	**1.48**	**3.46**
***Sphingomonadaceae***	***Proteobacteria***	**0.31**	**0.73**	**2.13**	**2.52**	**1.42**	**2.94**
*URHD0088*	*Proteobacteria*	1.50	1.65	0.93	1.26	1.33	91.35
***Mycobacteriaceae***	***Actinobacteriota***	**1.19**	**1.16**	**1.00**	**1.66**	**1.25**	**2.16**
***Caulobacteraceae***	***Proteobacteria***	**0.87**	**1.61**	**1.20**	**1.17**	**1.21**	**6.50**

Families that were significantly enriched among the AVSs that were shared between the two plants are indicated in bold text. The values under each sample type represent the average total RPKG of processes associated with phosphorus turnover. The rightmost column indicates the phylogenetic gain (PG) of each family. *RX* root (external).

To evaluate whether the retrieved MAGs exhibited an increased P turnover potential when compared to related genomes—suggesting adaptation to the *campos rupestres* substrates—we compared them to genomes from GTDB r89 regarding the number of genes involved in P turnover processes. Phylogenetic regression models revealed that six different processes were significantly enriched in the *campos rupestres* MAGs, including two types of phosphate transporters (the PiT and the ABC transporter families), inorganic pyrophosphatases and exopolyphosphatases (Fig. 5C).

**Fig. 6:**
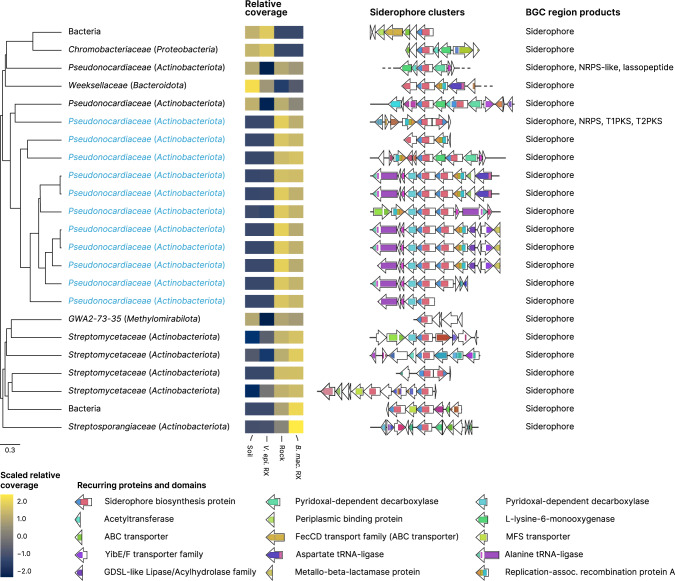
Structural diversity of siderophore biosynthetic gene clusters (BGCs) identified in the *campos rupestres* metagenomes. BGC regions containing siderophore clusters were hierarchically clustered using UPGMA with BiG-SCAPE distances. Groups of highly similar regions were identified based on their inconsistency coefficient and only the medoids are shown. Blue labels indicate the BGC regions that belong to the *Pseudonocardiaceae*-associated gene cluster clan. Taxonomies are presented at the family and phylum (in parenthesis) levels, except for two BGC regions whose contigs were assigned to the Bacteria domain. Heatmaps represent scaled means of log-transformed relative contig coverages in the four environments. Gene clusters are shown as arrays of genes (arrows) and their protein domains (colored blocks) centered at the siderophore biosynthesis protein. BGC regions containing other types of biosynthetic clusters (rightmost column) were trimmed to display only the loci assigned to the siderophore clusters.

We also evaluated the potential of the *campos rupestres* communities to mineralize P via siderophores. These molecules are synthesized by biosynthetic gene clusters (BGCs) and take part in P turnover by releasing P bound to iron cations [32, 33]. The assembled metagenomes had 42 siderophore-producing BGCs, which were grouped according to their domain organization and sequence similarity into 15 gene cluster families (GCFs) (Fig. 6). We found that a large gene cluster clan (GCC)—encompassing three GCFs and 18 siderophore BGCs (blue labels in Fig. 6) and assigned to the *Pseudonocardiaceae* family—possessed domains like those of desferrioxamine siderophores [34, 35]. This GCC, however, had distinctive domain organization and in tandem duplication of the siderophore biosynthesis protein (*IucA*/*IucC* family), indicating structural differences between their final products and described desferrioxamines. All siderophores within the *Pseudonocardiaceae* GCC were comparatively much more abundant in *B. macrantha* root and rock communities, which was supported by 16S rRNA gene data (Fig. 2C, Supplementary Fig. 4B), and similar abundance profiles were observed across most of the other siderophore BGCs. This difference in the siderophore synthesis potential was further supported by the fact that the “Biosynthesis of siderophore group nonribosomal peptides” KEGG pathway was significantly enriched in the *B. macrantha*-associated communities (FDR < 0.001).

Last, we evaluated the potential of symbiotic fungi to increase host P intake. Although arbuscular mycorrhiza are well known to increase P uptake by roots [36], *Velloziaceae* growing on severely P-impoverished substrates exhibit extremely reduced colonization by mycorrhizal fungi [8, 16, 37]. However, the ITS data showed that both plants harbored diverse endophytic fungi root communities (Supplementary Fig. 2A, B), which compelled us to interrogate whether the fungi associated with *V. epidendroides* and *B. macrantha* participate in P nutrition. Using a read-level targeted gene finding approach, we surveyed the metagenomic data to identify orthologs of the fungal high-affinity H^+^:Pi transporter (*PHO84*), which participates in both P uptake from the substrate and phosphate efflux to the plant in the symbiotic interface [38], which, in at least one case, is required for the establishment of endosymbiosis [39]. In total, 312 fungal *PHO84* were retrieved, 67.6% of which were assigned to taxa containing known endophytes, such as the *Dothideomycetes*, *Sordariomycetes*, *Leotiomycetes*, *Agaricomycotina*, and *Glomeromycetes* [40–43]. Analysis of their combined abundance revealed that, as expected for symbiotic fungi, *PHO84* was enriched in the rhizospheres relative to the adjacent substrates, albeit with low statistical significance (Supplementary Fig. 4C, LMM *p* value ≈ 0.18, *ω*^2^ ≈ 0.1).

### Reconstruction of the nitrogen cycle dynamics in the *V. epidendroides* and *B. macrantha* microbiomes

**Fig. 7:**
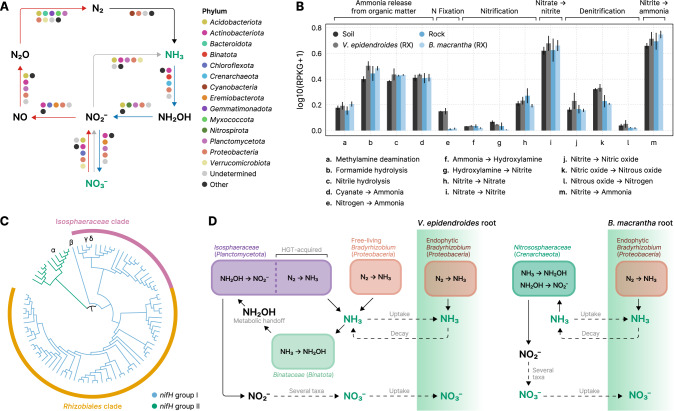
The N-turnover landscape of the *campos rupestres* microbiomes. **A** Phyla predicted to be involved in nitrogen-cycling reactions: fixation (black arrow), nitrification (blue arrows), denitrification (red arrows), and assimilatory nitrate reduction (gray arrow). Compounds that can be taken up by plants roots are depicted in green. Phyla that contributed less than 5% of the genes involved in each reaction were grouped under the “Other” category. **B** Total abundances (sum of RPKGs) of reactions involved in nitrogen turnover in the substrate and external root-associated communities. The abundances of multiprotein complexes (*nifHDK*, *amoABC*, *nxrAB*, *narGHI*, *napAB*, *nasAB*, *norBC*, *nirBD*, and *nrfAH*) were computed by averaging the abundances of their subunits. Vertical lines represent the standard error of the mean. RX = root (external). **C** Cladogram of phylogenies inferred from metagenomic *nifH* orthologs. Branches are colored according to the major group they belong to. The dominant clades are indicated by the outer rings. Orthologs encoded by MAGs encoding *nif* are indicated by greek letters: (α): *Verrucomicrobiota*, (β): *Enterobacteriaceae*, (γ and δ): *Isosphaeraceae*. **D** In the *V. epidendroides*-associated communities, nitrogen is mostly fixed by endophytic and free-living *Bradyrhizobium*, and by *Isosphaeraceae*, which likely received their *nif* complex via horizontal gene transfer (HGT). Ammonium is then oxidized into hydroxylamine by methylotrophic *Binataceae*. This molecule is then released from the cell and is oxidized into nitrite by *Isosphaeraceae*. In the *B. macrantha*-associated communities, nitrogen is likely converted into ammonium by endophytic *Bradyrhizobium*. Ammonia originated from organic matter decomposition is oxidized into hydroxylamine and, subsequently, into nitrite by *Nitrososphaeraceae*. In both plants, the oxidation of nitrite into nitrate is likely performed by a several taxa.

Bacteria and Archaea contribute to the nitrogen (N) cycle by participating in several steps such as the release of N from organic matter, N fixation into ammonia, nitrification, and denitrification (Fig. 7A). To understand the microbiome potential for N turnover in the *campos rupestres* and how they can impact plant nutrition, we evaluated the taxonomic profile and the abundances of genes involved in different steps of the nitrogen cycle (Supplementary Table 10). Among the highly abundant genes that are involved in nitrogen transformations we identified orthologs encoded by 14 different phyla (Fig. 7A, Supplementary Fig. 5A). Abundances of genes involved in cycling N were slightly higher in the rhizospheres of *V. epidendroides* and *B. macrantha* compared to their adjacent substrates (Fig. 7B, LMM *p* value ≈ 0.06, *ω*^2^ ≈ 0.02).

We set out to further survey the N fixation profile of the *campos rupestres* communities by probing the metagenomes and MAGs for genes encoding subunits of the nitrogenase complex (*nifHDK*). Sequence-based taxonomic assignment of nitrogenase subunits and phylogenetic analysis of the dereplicated set of all *nifH* subunits revealed that most of the *nif* diversity comes from the *Rhizobiales* order (*Proteobacteria* phylum, 55 out of 89) and the *Isosphaeraceae* family (*Planctomycetota* phylum, 22 out of 55) (Fig. 7C, Supplementary Fig. 5B). We also retrieved *nifH*-containing MAGs assigned to the *Enterobacteriaceae* family and *Verrucomicrobiota* phylum, the latter of which encoded a *nifH* from the group II clade, that is predominantly comprised of orthologs from obligate anaerobes [44].

Even though there is no report of an *Isosphaeraceae*-encoded *nif*, we found all subunits of the complex in four MAGs belonging to two species clusters, which persuaded us to reconstruct the evolutionary history of these orthologs. A phylogenetic analysis using *nifH* and *nifD* sequences from the *Isosphaeraceae* MAGs, other *Planctomycetota* species, and orthologs with high sequence similarity revealed that nitrogenase encoded by the MAGs formed a clade with *Gammaproteobacteria*, rather than *Planctomycetota*, strongly suggesting that they acquired *nifHDK* via horizontal gene transfer (HGT) (Supplementary Fig. 5C). Indeed, we found synteny between a *nif*-containing *Isosphaeraceae* contig and the *Pseudomonas stutzeri* genome, which harbors a packed *nif* cluster [45]. Additionally, gene-level taxonomic assignment revealed clear boundaries between the genes vertically inherited from ancestral *Isosphaeraceae* and genes within the region received via HGT (Supplementary Fig. 5D).

As for the *Rhizobiales*, the vast majority (≈91.5%, or 54 out of 59) of the orthologs prior to dereplication were found in unbinned contigs from *V. epidendroides* and soil metagenomes, resulting in an apparent major difference between the N fixation potential of the communities associated with the two plants. As the metagenomic data mostly captured free-living populations and because endophytic bacteria are major players in the N fixation process, we examined the abundances of *Bradyrhizobium*—a genus within the *Rhizobiales* that encompasses several endophytic diazotrophs—using 16S rRNA gene data and found that these bacteria were enriched in the endophytic compartments of both plants (Supplementary Fig. 5E). Due to the lack of metagenomes assembled from endophytic root communities, we employed PCR to confirm the presence of the *nifH* gene in the microbiome DNA extracted from the roots of six samples of each plant species (Supplementary Fig. 5F). Additionally, we employed sensitive read-level gene identification to probe *Bradyrhizobium nifH* sequences in metagenomic data and found that these orthologs were present in the *B. machantha* and rock metagenomes, albeit in lower levels than in the *V. epidendroides*-associated communities, and that they were enriched in the rhizospheres (Supplementary Fig. 5G, LMM *p* value < 0.05; *ω*^2^ ≈ 0.45). We also identified two *Bradyrhizobium* contigs, retrieved from the metagenomes of both plants, that harbored proviral sequences containing the *exoZ* gene, which encodes a cell surface modifying acetylase that increases the efficiency of endosymbiosis establishment [46] (Supplementary Fig. 5H). Comparison with IMG/VR’s [47] viral genomes revealed that similar bacteriophages of the *Caudoviricetes* class were previously detected in root nodule and rhizosphere communities.

Besides the ammonia generated by N fixation, the primary source of inorganic nitrogen to plants is nitrate, produced through sequential oxidation of ammonia (Fig. 7A). These reactions are carried out by ammonia-oxidizing Bacteria (AOB) and Archaea (AOA), which form nitrite in two steps (converting ammonia into hydroxylamine, and hydroxylamine into nitrite), and by nitrite-oxidizing bacteria, which convert nitrite into nitrate (Fig. 7A). Inspection of the taxonomic assignments of contigs containing ammonia monooxygenase (*amoABC*) and hydroxylamine oxidoreductase (*hao*) revealed that the *Proteobacteria*, which encompass all the traditional AOB [48], are a minor fraction of the communities with potential for nitrification of the *campos rupestres* (Supplementary Fig. 5A).

Canonically, both steps of nitrite formation are carried out by bacteria harboring both *amoABC* and *hao*; however, we found that the taxonomic profiles of these two enzymes were contrasting (Supplementary Fig. 5A). Inspection of the MAGs’ gene repertoires revealed that no genome encoded both the *amo* complex and *hao* (Supplementary Fig. 5H). Two groups of ammonia-oxidizing MAGs were retrieved: the *Nitrososphaeraceae*, a family of AOA that harbors *amoABC* and is known to oxidize hydroxylamine into nitrite without *hao* [49]; and the *Binataceae*, a family of the methylotrophic phylum *Binatota* which encodes the methane monooxygenase cluster (*pmoABC*), a close ortholog of *amoABC* that has been shown to oxidize ammonia [50]. As for *hao*, we found highly abundant *Isosphaeraceae* MAGs encoding orthologs that contained all the 16 heme-binding cysteines necessary for function (Supplementary Fig. 5I).

## Discussion

Here we show that the patterns observed in the sequencing data of the microbiomes associated with *V. epidendroides* and *B. macrantha* are compatible with our initial hypothesis that these species form dynamic associations with diverse microbial communities that promote nutrient turnover. First, community composition analysis revealed that the *campos rupestres* microbial communities are diverse and significantly novel. Second, the microbiomes associated with *V. epidendroides* and *B. macrantha* are significantly distinct and some of the differences can be explained by environmental factors, such as low carbon availability in the rocks. Furthermore, the communities common to both species were found to be highly abundant, suggesting that they are highly adapted to the nutritional limitations of the environment. Third, there is an enrichment of genes involved in organic compound intake, P turnover, and N turnover in the root surface, which is expected under a model of recruitment of beneficial microorganisms. Last, the shared microbiota was found to be involved in both P turnover and N fixation. Some of the observed differences in nutrient turnover dynamics, such as siderophore production and N fixation by free-living microorganisms, could be attributed to the contrasting properties of the two substrates that were investigated.

### The *campos rupestres* microbial communities harbor a high degree of taxonomic novelty

Because the *campos rupestres* constitute an underexplored biodiversity hotspot with prominent levels of plant endemism, we evaluated the uniqueness of the communities associated with *V. epidendroides* and *B. macrantha* using different approaches. Using the newly devised WACI metric we showed that both the prokaryotic and fungal communities associated with the investigated *Velloziaceae* are highly novel. Retrieved MAGs expanded the phylogenetic diversity of understudied groups (Fig. 1E), including the *Eremiobacterota*, for which we demonstrated potential for phosphorus turnover (Table 1) and carbon fixation (Supplementary Fig. 3E, F), the *Dormibacterota*, also involved in carbon fixation (Supplementary Fig. 3F), and the *Binatota*, a group of methylotrophic bacteria [51] that likely participates in nitrogen cycling in the *campos rupestres* soils (Fig. 7D, Supplementary Fig. 5A, H).

### The microbiomes of *V. epidendroides* and *B. macrantha* are highly differentiated but share a core of highly efficient colonizers

The different microbiomes of the two plants, despite their geographic proximity [17], suggests that contrasting substrates and host biology shape plant-associated communities in the *campos rupestres*. Furthermore, the finding that microbiome specialization is driven by taxonomic biases indicates host and environmental pressures select specific functions. One notable example of such structured differentiation is the recurrent enrichment of *Actinobacteriota* families in *B. macrantha*, which can be attributed to the resilience of these bacteria to low humidity [52] and influenced the siderophore production in the rock-dwelling communities.

Despite microbiome specialization, the two plants shared a core microbiota of efficient microbial colonizers that are likely adapted to the harsh environmental conditions of the *campos rupestres*. Accordingly, 80% of the bacterial families with the highest total abundance of genes involved in phosphorus turnover were enriched among the shared ASVs (Table 1, Fig. 3B), corroborating our hypothesis that core taxa have genetic potential to increase plant fitness and might be selected by both plants. Additionally, we found that, despite not being detected in rock metagenomes, N-fixing *Bradyrhizobium* were shared by both plants as they could form endophytic associations in the root tissue.

Even though the rocks over which *B. macrantha* grows are nutrient depleted as compared to the soils [17]—especially regarding organic matter and nitrogen—we found that the prokaryotic diversity did not significantly differ between the microbiomes of the two plants (Supplementary Fig. 1B), suggesting that the rock-dwelling microorganisms possess mechanisms that allow them to grow under severe nutritional limitations. Indeed, we found evidence that potential for phosphorus and nitrogen turnover were not reduced in the rocks. In contrast, fungal communities associated with *B. macrantha* were less diverse than the ones associated with *V.epidendroides*, which is in accordance with previous reports that plants growing over rocks have reduced fungal colonization in the *campos rupestres* [8].

### The core *Velloziaceae* microbiome harbors potential for phosphorus turnover

Species of the *Velloziaceae* family increase P availability in soils [16] and rocks [6] by secreting exudates containing carboxylates that release phosphate bound to cations, such as iron and aluminum [53]. Despite these adaptations, some forms of P are unavailable for plant uptake and are only accessed by bacteria, which harbor diverse processes that make P bioaccessible [27]. As root exudates are rich in organic compounds that recruit and sustain microbial communities [20], we hypothesized that *Velloziaceae* exudates fulfill a dual role: they increase the labile P concentration by direct solubilization and also recruit microorganisms that possess a complementary repertoire of molecular processes that increase P availability in the root’s vicinity. In this scenario, microorganisms would use phosphate for their own needs, but would benefit the system in the long term by mobilizing P otherwise unavailable to plants [28] (Fig. 5A).

We assessed capability for active recruitment of microorganisms by evaluating the abundances of transporters with specificity for amino acids and organics acids that are secreted by roots [25, 26]. The increased abundances of these transporters in the rhizosphere communities of both plants (Fig. 4) suggests that microorganisms able to take up organic compounds display enhanced fitness in the vicinity of the roots, which is consistent with our hypothesis that *Velloziaceae* root exudates shape the plant microbiome. We highlight that accumulation of non-exudated organic molecules in the rhizosphere could also explain the observed transporter enrichment, therefore experimental validation will be necessary to confirm whether these transporters can intake root exudates and to determine if they mediate a microbiome recruitment by the plants.

The enrichment of processes linked to P turnover in the root microbiomes compared to the substrates indicates that the plant-associated bacteria have genetic potential to increase bioaccessible P (Fig. 5B). Furthermore, we also observed that the exopolyphosphatase activity and the catabolism of phosphanates, which are not used by plants to increase P uptake, are abundant in the root communities, indicating a complementarity between repertoires of the hosts and their associated microbiota (Fig. 5A). The fact that 80% of the taxa with elevated P turnover potential were enriched within the microbiota shared between *V. epidendroides* and *B. macrantha* suggests that both plants associate with a common set of microorganisms that might play a part in P mobilization and are possibly recruited. To illustrate this scenario, we show that the *Bryobacteraceae*, whose known members are acidophilic chemoheterotrophs that consume organic acids [54], were enriched within the shared associated microbiota, had high abundances of genes involved in P turnover, especially in the rhizospheres (Table 1). Other five shared families with elevated P-mobilization potential (*Solirubrobacteraceae*, *Beijerinckiaceae*, *Acidobacteriaceae*, *Mycobacteriaceae*, and *Caulobacteraceae*) were previously found to be associated with low P soils [23], supporting the hypothesis that the shared microbiota consists of highly adapted lineages. The participation of these taxa in P turnover in the *campos rupestres* and their recruitment still need to be confirmed experimentally in future studies.

By evaluating the production of siderophores by plant-associated bacteria, we found that the examined communities harbored structurally diverse BGCs, including some that form hybrid regions with other classes of biosynthetic clusters (Fig. 6, rightmost column), which suggests a diversified repertoire of final siderophore products. In addition, the siderophore repertoires of *B. macrantha*-associated communities were much larger, which could be interpreted as an indirect consequence of the higher abundance of the BGC-rich [55] *Actinobacteriota* in these microbiomes. We consider, however, that a complementary nutrient-driven explanation is also appropriate: as the iron content of the sampled rocks is around 8-fold lower than that of the soils [17] this nutrient is under increased demand in *B. macrantha* microbiomes, increasing the fitness of *Actinobacteriota* populations that are efficient at scavenging iron and, consequently, solubilizing phosphate.

Despite previous reports suggesting that *Velloziaceae* growing under severe P-impoverishment exhibit reduced fungi colonization [8, 16, 37] we found an enrichment of the *PHO84* phosphate transporter, encoded by endosymbiotic fungi, in the rhizosphere (Supplementary Fig. 4C). Given that the below-ground fungal communities under investigation included highly novel fungal lineages (Supplementary Fig. 1B), we hypothesize that undescribed species may establish associations with the radicular systems of both plants, occupying the ecological niche left by arbuscular mycorrhiza whose abundances were extremely reduced in the *campos rupestres* [7, 16, 56] (median *Glomeromycota* ASV abundance in below-ground samples: 0.23% and 0.20% in *V. epidendroides* and *B. macranta*, respectively).

### Nitrogen cycling is heavily influenced by the substrate and involves newly described microbial lineages and interaction dynamics

In the *campos rupestres*, nitrogen is constantly lost from the biological pool due to seasonal fires [1]. Therefore, as biological fixation of atmospheric N is pivotal to sustain the biomass of this ecosystem, we set out to investigate the N cycle in the context of the *V. epidendroides* and *B. macrantha*-associated communities. Our finding that genes involved in N-transforming processes in the *campos rupestres* are encoded by several taxa challenges the traditional view that some reactions, such as ammonia and nitrite oxidation, are carried out by few restricted clades [57, 58]—although experimental validation is necessary to confirm the activity of the detected genes. Additionally, as we observed for genes involved in P-turnover, we found that N cycle processes were also enriched in the rhizosphere, although at a much lower level, suggesting that microorganisms that can perform some of the transformations in the N cycle have increased fitness when associated with plants.

We retrieved four *Isosphaeraceae* MAGs with metabolic potential for nitrogen fixation from *V. epidendroides*-associated communities. Enrichment of this family in the microbiomes of *V. epidendroides* was also observed in ASV data (Fig. 2C). Although *nifHDK* genes have been documented in some marine *Planctomycetota* [59], their complexes belong to the group II *nif*, while the *campos rupestres Isosphaeraceae* orthologs belong to group I (Supplementary Fig. 5B, C). A combination of phylogenetic reconstruction and synteny analysis indicates that the N fixing potential of the *Isosphaeraceae* MAGs was the result of a HGT from a *Gammaproteobacteria* (Supplementary Fig. 5C, D). Given that these MAGs compose a large fraction of the *campos rupestres nifH* diversity (Fig. 7C, Supplementary Fig. 5B) and that there is prior evidence for *nif* HGT events [45, 60–62], we argue that horizontal transmission of the nitrogenase activity can affect the biogeochemical N cycle and plant nutrition.

We also found 59 *nifH* orthologs that were assigned to the *Rhizobiales* order, a lineage that comprises several known diazotrophs. Only five of these orthologs were retrieved from *B. macrantha* and rock metagenomes and, although several *Bradyrhizobium* MAGs were recovered from these environments, none had the metabolic potential to fix N. We hypothesize that the lack of *nif* is an advantageous trait for rock-dwelling bacteria, as the N fixation reaction is very carbon-demanding [63] and could cripple their growth in a carbon-poor environment [17]. Accordingly, we found evidence that endophytic diazotrophs, which are not limited by carbon, are present in the roots of both species (Supplementary Fig. 5F, G). In addition, the finding that virus-encoded *exoZ* is integrated into *Bradyrhizobium* genomes (Supplementary Fig. 5H) further supports that these bacteria establish endophytic associations, because phages employ auxiliary metabolic genes to enhance host fitness [64–66].

Regarding nitrification, we found no evidence for the participation of canonical AOB that possess the molecular machinery for the two-step ammonia conversion into nitrite within a single cell. Instead, we found that the ammonia and hydroxylamine oxidation reactions in the soil are potentially carried out by distinct populations belonging to the *Binataceae* and *Isosphaeraceae* families, respectively. Even though *hao* has no other known function besides hydroxylamine oxidation, *amoABC*/*pmoABC* can oxidize both ammonia and methane. In fact, some methylotrophs use *hao* as a hydroxylamine detoxification mechanism [50]. However, no *Binatota* genome (22 genomes in GTDB r89 and 86 genomes in GEM [67]) encode *hao*, which lead us to hypothesize that the methylotrophic *Binataceae* use metabolic handoff associations [68] with *hao*-encoding *Isosphaeraceae* as a means of hydroxylamine detoxification, benefiting the recipient cell by providing them with an energy-rich molecule. All the *Isosphaeraceae* with potential for N fixation also contained *hao*, suggesting that these genomes fulfill a dual role in the nitrogen cycle. Furthermore, *Isosphaeraceae* genomes in GTDB r89 encoded *hao*, indicating that ancestral lineages already had potential for N turnover.

As we investigated a broader genomic dataset, we found that the decoupling of the *amoABC*-*hao* system was commonplace, as approximately half (92 out of 193) of *hao*-containing bacterial genomes in GTDB (release 89, AnnoTree [69] annotation) have no subunit of the *amoABC* complex, including 16 *Planctomycetota* and 9 *Acidobacteriota* genomes (both phyla encoding *hao* in our metagenomes). Further strengthening our hypothesis, the alternative mechanism of hydroxylamine detoxification using the cytochrome P460 [70] was also absent in all *Binatota* MAGs and was found in 6 of 7 *Isosphaeraceae* genomes. However, even though hydroxylamine is known to be released from the cell to the environment [71, 72], it is a very reactive molecule, being readily oxidized into nitrogen trace gases [73, 74]. Therefore, close physical proximity is presumably necessary for this putative metabolic handoff to be effective.

Altogether, our results suggest that most of the N fixation in *V. epidendroides* is performed by *Isosphaeraceae* and *Bradyrhizobium*, both free-living and endophytic. In *B. macrantha*, due to nutritional limitations of its substrate, most fixation is performed by endophytic communities, mostly comprised of *Bradyrhizobium* (Fig. 7D, Supplementary Fig. 5I). The importance of *Bradyrhizobium* for the plants is supported by the fact that its family (*Xanthobacteriaceae*) had the highest level of enrichment among the ASVs that were shared between the root communities (Fig. 3B). As for nitrification, our MAG data suggest that potential for ammonia oxidation is primarily found in *Binataceae* (in *V. epidendroides*) and in *Nitrososphaeraceae* (in *B. macrantha*), with a critical metabolic handoff interaction between *Binataceae* and *Isosphaeraceae* to convert hydroxylamine into nitrite (Fig. 7D). Although other taxa, such as *Acidobacteriota* and *Proteobacteria*, also may be involved in these reactions, we found no evidence for the participation of canonical AOB.

## Conclusions

This study set out to characterize the composition and functions of the microbiomes of two plant species that grow in the severely nutrient-impoverished substrates of the Brazilian *campos rupestres*. The results are consistent with our hypothesis that these plants establish close association with diverse microorganisms, including a shared set of highly efficient colonizers, and that these microbial communities harbor genetic potential for carbon, phosphorus, and nitrogen turnover. We note, however, that experimental data is necessary for direct confirmation of microbiome recruitment and microorganism-driven nutrient turnover. Taken together, our findings highlight that plant-associated microorganisms encode processes that can contribute to plant nutrition and that assessing microbial diversity is crucial to understand the dynamics of nutrient cycling. We propose that future research considers the microbial diversity and ecology and their contribution to develop holistic models of plant fitness in nutrient-limited environments such as the *campos rupestres*.

## Methods

### Sample collection and metagenomic sequencing

The study design, sampling methodology, library preparation, and sequencing that were used in this study were thoroughly detailed previously [17]. Briefly, substrate (soil and rock), root, stem, and leaves were sample from six individuals of *V. epidendroides* and *B. macrantha* in March of 2017, at the end of the wet season. For each plant species, a sampling area of approximately 200 m^2^ was defined and six individuals were selected and assigned random identifiers from R1 to R6. For *V. epidendroides*, the soil surrounding the plant within a 20 cm radius was excavated to a depth of 15 cm. For *B. macrantha*, the adjacent rocks were fragmented until roots were exposed and the pieces of rock were collected and further grinded to small pieces. Host-associated microbiota were gathered from the external and internal compartments of the root, stem, and leaves of the sampled plants using the methods described in [17] for isolating the epi and endophytic communities.

Environmental DNA was extracted using the DNeasy PowerSoil Kit (Qiagen, Hilden, Germany). Amplicon sequencing of the 16S rRNA gene V4 region and the internal transcribed spacer 2 (ITS2), for profiling prokaryotic and fungal communities, was achieved through PCR amplification of the DNA extracted from samples of all six individuals (substrates and the external and internal plant organs) using the 515FB/806R [75] and ITS9_Fwd/ ITS4_Rev [76] primer pairs and subsequent sequencing using the MiSeq System (Illumina) platform to generate 2 × 300 bp reads. Metagenomic libraries of the external root and substrate communities from three individuals (samples R1 to R3) were generated with the HiSeq System (Illumina) sequencing platform, yielding 2 × 150 bp reads.

### Amplicon sequence variants inference and taxonomic assignment

Sequencing reads of 16S rRNA gene and ITS were processed to remove the PCR primer sequences with cutadapt [77] (version 1.16). We retained read pairs where complete sequences of both the forward primer in the R1 read and the reverse primer in the R2 read were detected. Amplicon sequence variants (ASV) inference was then performed separately for the 16S rRNA gene and ITS libraries using DADA2 [78] (version 1.6.0). For the 16S rRNA gene libraries we truncated the reads to remove bad-quality regions (245 bp for R1 and 180 bp for R2). ITS reads were not truncated to a fixed length to facilitate overlap between the read pairs as this region presents significant length variation across genomes. Next, reads with ambiguous bases or with an expected number of errors greater than two were filtered out and DADA2’s error models were fitted to the R1 and R2 reads of both types of amplicon. These models and the dereplicated reads pooled from all samples were used to infer and correct sequencing errors. ASVs were obtained by merging read pairs that overlapped at least 16 bp and removing putative PCR chimeras. Finally, we excluded possibly spurious ASVs from downstream analysis by filtering out the ASVs that were observed in a single sample [79].

Taxonomic assignment of ASVs was performed with the IDTAXA algorithm [80] (part of the DECIPHER library, version 2.8.1) using a confidence threshold of 40% and the GTDB [81] (release 89) and UNITE [82] (Feb. 2020 release) databases as taxonomic references for 16S rRNA gene and ITS sequences, respectively. IDTAXA was also employed to identify ASVs derived from organellar genomes by assigning 16S rRNA gene sequences to taxa from the SILVA database [83] (release 138), which includes ribosomal genes of mitochondria and chloroplast. ASVs found to be derived from organellar genomes were excluded from downstream steps.

### Investigation and statistical analyses of ASV data

Community alpha diversity, quantified as the richness and evenness, was computed from 16S rRNA gene and ITS ASV count data using the vegan library (version 2.5-5). Richness was estimated from count matrices rarified to 5,000 reads, a value that was chosen because it is close to the lowest sequencing depth among the samples, which allowed all the samples to be included. Robustness of the results to rarefaction was assessed by confirming that the conclusions did not change when rarefying count matrices to values up to 30,000. Community evenness was assessed through the Pielou’s equitability index computed from non-rarefied read counts. To test for relationships between the host plant species and community alpha diversity, the data were modeled using linear mixed-effects models (LMMs) with the lmerTest package (version 3.1-0) as follows: *Y* = *β* × *H* + *S* + *C* + *E*, where *β* is the regression coefficient, *H* encodes the host species, *S* and *C* are the random effects of the sampled individual and the sample type, respectively, and *E* is a vector of errors. The sample types used for modeling were the external and internal communities of the root, stem, and leaves; substrate samples were not included.

Beta diversity was appraised using Bray–Curtis and weighted UniFrac dissimilarities computed from relative ASV abundances using the phyloseq library [84] (version 1.34.0). The phylogenetic trees used to compute weighted UniFrac were reconstructed using IQ-TREE [85] (version 2.0.3, parameters: ‘--fast -m GTR+G4’) from 16S rRNA gene and ITS ASV alignments generated with MAFFT [86] (version 7.464, parameters: ‘--auto’). The statistical significance of grouping communities according to their host plant within the Bray–Curtis and UniFrac spaces was estimated using PERMANOVA ('adonis' function from vegan) [87].

The differentiation of communities belonging to the same type (substrate and the external/internal communities of the root, stem, and leaves) but associated with different host plants was appraised using two approaches. First, the main taxa driving the differentiation between *V. epidendroides* and *B. macrantha*-associated communities were identified by using ALDEx2 [88] (version 1.18.0, parameters: ‘denom = “zero”, mc.samples = 1000’) to rank ASVs by their effect size. Then, the hypeR [89] library (version 1.6.0) was employed to identify statistically significant (FDR ≤ 10^−5^ and score ≥ 0.2) family-level taxa enrichment in each host plant by performing Kolmogorov–Smirnov tests on the ordered ASV lists. Second, we determined the relative amount of ASVs that were shared between pairs of communities associated to different hosts. Shared ASVs were defined as ASVs that were observed in at least two replicates within each of the communities being compared. Family-level taxa enrichment in the sets shared ASV was evaluated using hypergeometric tests (FDR ≤ 10^−3^), as implemented in hypeR.

To measure the taxonomic novelty of each community we devised the weighted average community identity (WACI) metric. Specifically, ASV sequences were aligned to reference databases (GTDB and UNITE for 16S rRNA gene and ITS sequences, respectively) using BLAST [90] (version 2.9.0) and the WACI was computed as the average alignment identity to the best hit weighted by the relative ASV abundance in the sample. Differences between the WACI of below-ground (substrate and root-associated) and above-ground (stem and leaves-associated) communities were evaluated using LMMs as follows: *Y* = *β* × *R* + *S* + *C* + *E*, where *β* is the regression coefficient, *R* encodes the region (above or below-ground), *S* and *C* are the random effects of the sampled individual and the sample type, respectively, and *E* is a vector of errors.

### Metagenome assembly and retrieval of metagenome-assembled genomes

Metagenome sequencing reads from 12 different samples (3 soil, 3 rock, 3 *V. epidendroides* rhizosphere, 3 *B. macrantha* rhizosphere) were quality-trimmed using cutadapt [77] (version 1.16, parameters: ‘--pair-filter=any -m 25 -q 5,5’). To recover unpaired reads that were discarded because their pairs did not satisfy the length threshold, cutadapt was executed again on these pairs using only the ‘-m 25 -q 5,5’ parameters. Trimmed read pairs and the unpaired reads were used for metagenomic assembly with MEGAHIT [91] (version 1.2.7, parameters: ‘--k-min 27 --k-max 147 --k-step 10’). Additionally, four co-assemblies were generated by pooling the reads from the three samples of each of the four sample types (soil, rock, and the rhizospheres of each plant) and assembling them together. Contigs shorter than 500 bp and 1,000 bp were filtered out from the individual and co-assemblies, respectively. Taxonomic assignment of the resulting contigs was attained with MAGpurify2 (available at https://github.com/apcamargo/magpurify2), using a database based on GTDB release 89 (doi: 10.5281/zenodo.3817702). We also performed protein-level assembly with PLASS (version 2.c7e35) [92], using the trimmed read pairs as input. Only peptides containing both start and stop codons and at least 60 amino acids were retained.

Metagenome-assembled genomes (MAGs) were obtained by binning the contigs of each assembly and then dereplicating the bins recovered from assemblies of the same environment. First, the contig coverage across all conditions was obtained using Bowtie 2 [93] (version 2.3.5) to cross-map the reads of each sample to each assembly. Next, the read mappings were employed to bin contigs longer than 2 kb using four different binning tools: MetaBAT2 [94] (version 2.14), MaxBin2 [95] (version 2.2.7), CONCOCT [96] (version 1.1.0), and Vamb [97] (version 3.0.1). DAS Tool [98] (version 1.1.2) was then executed to aggregate the four sets of bins generated from each assembly into 16 non-redundant bin sets. To dereplicate near-identical genomic bins, we used Galah (version 0.1.0) to cluster genomes with ≥ 99% average nucleotide identity (ANI) across the four sets of bins generated from each sample type (three individual assemblies and one co-assembly) and select the best genome within each cluster based on completeness and contamination estimates obtained with CheckM [99] (version 1.1.3). MAGpurify2 was then executed to remove putative contaminant contigs within each bin. Last, the final set of bins (hereinafter referred to as MAGs) was attained by selecting the medium and high-quality MAGs, according to the MIMAG standard [100]. MAG abundance was estimated using CoverM (version 0.3.2, available at https://github.com/wwood/CoverM, parameters: ‘--methods mean --min-read-percent-identity 0.95’).

### Taxonomic assignment, species clustering and phylogenetic novelty of MAGs

MAGs were assigned to GTDB r89 taxa using the phylogenetic placement algorithm implemented in GTDB-Tk [101] (version 1.1.0). In addition, MAGs were grouped into *de novo* taxonomy-independent species clusters through pairwise genome comparisons. Briefly, species clusters were iteratively constructed by grouping pairs of MAGs whose alignment covered at least 65% of the length of the shorter genome with at least 95% ANI [21, 102, 103], according to FastANI [21] (version 1.32) estimates. For each cluster, the highest scoring MAG (score was defined as ‘completeness – 5 × contamination’) was chosen as an operational species representative.

The amount of phylogenetic novelty brought by the *campos rupestres* MAGs to different bacterial and archaeal taxa at varying taxonomic ranks (phylum, class, order, family, and genus) was quantified using the phylogenetic gain metric [104], which represents the branch length added by a subset of genomes to a specific clade in a phylogeny. To compute this metric, the multiple sequence alignments of bacterial and archaeal markers generated by GTDB-Tk were used to reconstruct maximum-likelihood phylogenetic trees with IQ-TREE (version 2.0.3, parameters: ‘--fast -m WAG+G’). Branch lengths within each evaluated taxon were measured with DendroPy [105] (version 4.4.0).

### Functional annotation and protein clustering

Functional annotation was achieved by assigning KEGG orthologs (KO) [106], Pfam families [107], and TIGRFAM families [108] to protein sequences obtained from the metagenomic assemblies, PLASS protein-level assemblies, and MAGs. Metagenomic assemblies were annotated using the DOE-JGI IMG Annotation Pipeline [109, 110] (v.5.0.15). Protein-level assemblies obtained with PLASS were annotated using KofamScan [111] (KOfam release 2020-12-08, parameters: ‘--e-value 0.01’), a modified version of PfamScan (available at https://github.com/apcamargo/hpc_pfam_search) to assign Pfam (release 33.1) families, and hmmsearch to assign TIGRFAM (release 15.0) families (parameters: ‘--cut_nc’). MAGs were annotated using a modified version of EnrichM (version 0.5.0, available at https://github.com/apcamargo/enrichM), manually modified to update the Pfam (release 33.1) and KEGG (release 2020-05-10) databases and to use pre-established thresholds (‘--cut_ga’ for Pfam, ‘--cut_nc’ for TIGRFAM). Transporters were identified by aligning metagenomic proteins to reference transporters of the TCDB database [112] (release 2021/04/07) using MMseqs2 [113] (release 13-45111, search parameters: ‘-e 1e-5 --min-seq-id 0.3 -c 0.7 -s 7.5’) and extracting the best hit for each query. Carbohydrate active enzymes (CAZy) were identified by searching protein sequences against dbCAN [114] (version 9) profiles using hmmscan and then filtering the results with dbCAN’s hmmscan-parser script (thresholds: alignment coverage ≥ 35%, E-value ≤ 1e-18).

### Read-level gene identification

We used GraftM [115] (version 0.13.1) to identify target genes from read sequences by comparing them to custom protein databases. The database containing orthologs of the *Saccharomyces cerevisiae* high-affinity phosphate:H^+^ symporter (*PHO84*) was built from all UniProt (release 2020_06) protein sequences annotated with the TIGR00887 TIGRFAM. For the *nifH* database, we retrieved all genes annotated with the K02588 KEGG ortholog from AnnoTree [69] (doi:10.5281/zenodo.3732466). Manual intervention was required to root the phylogenetic tree using phytools (version 0.7–70) ‘midpoint.root’ function.

### MAG metabolic potential inference

Due to the inherent incompleteness of MAGs, it is challenging to ascertain their underlying functional repertoire [116]. Thus, to determine the MAG metabolic potential we established multiple criteria based on the presence/absence of key genes, the completeness of multiprotein complexes, and the fraction of genes in KEGG modules. Specifically:

**Photosynthesis**: at least one protein of a photosynthetic reaction center (annotations: K02689, K02690, K08928, K08929, K02703, K02706, K08940, PF00223, PF00124).

**CBB cycle**: at least 60% of the metabolic steps in the KEGG module (M00165) and at least one Rubisco subunit (annotations: K01601, K01602).

**Aerobic CO oxidation**: encode the catalytic subunit of the aerobic carbon monoxide dehydrogenase (*coxL*) (annotation: TIGR02416).

**Nitrogen fixation**: at least two subunits of the *nifHDK* complex (annotations: K02588, K02591, K02586).

**Ammonia oxidation**: at least two subunits of the *amoABC*/*pmoABC* complex (annotations: K10944, K10945, K10946).

**Hydroxylamine oxidation to nitrite**: presence of the *hao* and cytochrome c554 (necessary for proper *hao* function [117]) genes (annotations: K10535, PF13435). As Archaea can produce nitrite from ammonia without a *hao* ortholog [49], we considered that all archaeal MAGs capable of ammonia oxidation were also able to oxidize hydroxylamine.

**Hydroxylamine oxidation to nitrous oxide**: encode at least one gene containing the Cytochrome P460 domain (annotation: PF16694).

### Gene abundance estimation

To quantify gene abundance across all samples, we first used Linclust to cluster genes that were identical at the nucleotide level (parameters: ‘--cluster-mode 2 --cov-mode 1 --min-seq-id 1.00 -c 0.9 --kmer-per-seq 80’). Next, Salmon (version 1.4.0) [118] was executed to map the reads from each of the 12 metagenomic samples to the dereplicated genes and estimate read counts and effective lengths (parameters: ‘--meta’). Last, we quantified gene abundance using the RPKG (reads per kilobase per genome equivalent) metric so that the values are proportional to the expected gene copy number per cell [119]. To do that, we first estimated the average genome size of each sample using MicrobeCensus [119] (version 1.1.1) and then determined the number of genome equivalents (GE) as (*Sample read count* × 300)/*Average genome size*, where 300 corresponds to the total length of each read pair. Next, RPKG was computed using the following formula: (*Mapped reads* × 1,000)/(*Effective length* × *GE*).

### Functional comparison between metagenomes

We used RPKG values to evaluate differences in the carbon, phosphorus, and nitrogen turnover potential between the substrate and root-associated communities. We first summed the RPKGs of genes associated with the same biological process and then evaluated for systematic differences in abundance between the contrasted conditions using LMMs: *log*10(*RPKG* + 1) = *β* × *N* + *H* + *P* + *E*, where *β* is the regression coefficient, *S* is the environment (substrate or root), *H* and *P* are the random effects of the host plant species and the biological process, respectively, and *E* is a vector of errors.

Gene abundance data were also used to contrast the microbiomes of *V. epidendroides* and *B. macrantha* by identifying KEGG modules and pathways that were significantly enriched in the communities associated with one plant relative to other. To do that, we first translated and then clustered the dereplicated gene set at 90% sequence identity (parameters: ‘--cluster-mode 2 --cov-mode 1 --min-seq-id 0.90 -c 0.9 --kmer-per-seq 80’). The protein cluster membership information was then used to aggregate Salmon’s gene-level estimates into cluster-level abundances using tximport’s [120] (version 1.18.0) ‘scaledTPM’ approach. Cluster-level abundances were then imported into a DESeq2 [121] (version 1.30.0) object and between-sample normalization was performed by setting the sample’s size factor as the ratio between the number of GEs in the sample and the median of the number of GEs across all samples. This normalization allowed subsequent statistical tests to effectively compare the expected number of gene cluster copies per cell, a biologically meaningful quantity [119, 122].

Highly differentially abundant gene clusters were identified using a Wald test with strict parameters (s-value ≤ 0.005; DESeq2 options: ‘altHypothesis = “greaterAbs”, lfcThreshold = 0.5’). Significant enrichment of KEGG modules and pathways (FDR ≤ 0.05, score > 0) in the communities associated with each plant was appraised using hypeR’s Kolmogorov–Smirnov test on lists of gene clusters ranked by apeglm-shrunken [123] log2 fold changes. For this analysis, we assigned to each gene cluster the KOs of all the genes within it and then assigned KEGG modules and pathways according to the KO-to-module and KO-to-pathway relationships retrieved using KEGG’s REST API (release 97, 2021/01).

### Protein family copy number comparison between MAGs and GTDB genomes

To appraise genomic adaptations of bacteria from the *campos rupestres* to P limitation, we employed phylogenetic regressions to test whether MAGs retrieved from *campos rupestres* communities have significant differences regarding the number of genes involved in P turnover processes. To account for differences in genome size, we decided to use the genic density (number of genes per Mb) as the response variable using the following formula: *N* × 10^6^ / *Genome size* = *β* × *CP* + *E*, where *N* represents the number of genes involved in P turnover processes in the genome, *CP* represents a binary predictor variable that indicates whether the genome was retrieved from the *campos rupestres*, and *E* is phylogenetic covariance-aware vector of errors. The models were created using the ‘phylolm’ function (parameters: ‘model = “lambda”) of the phylolm [124] package (version 2.6.2).

Functional annotations for all GTDB (release 89) bacterial genomes were retrieved from AnnoTree. To avoid biases that could arise due to different annotation methods used for KEGG ortholog assignment by AnnoTree and our MAG annotation pipeline, we reannotated our MAGs using EnrichM’s UniProt-based KO assignment with the same thresholds used by AnnoTree.

### Biosynthetic gene cluster identification and clustering

We used antiSMASH [125] (version 5.1.2) to identify biosynthetic gene clusters (BGCs) in our metagenomes, ignoring contigs shorter than 5 kb. We then used BiG-SCAPE [126] (version 1.0.1, parameters:‘--cutoffs 0.5 --clan_cutoff 0.5 0.7 --mibig’) to cluster BGCs into gene cluster families (GCFs), and then cluster GCFs into gene cluster clans (GCC).

To investigate the structural diversity of siderophore-producing BGCs we used BiG-SCAPE (parameters: ‘--cutoffs 1.0’) to generate an all-versus-all distance matrix of all regions containing a IucA/IucC domain (PF04183). Next, these BGCs were clustered using the UPGMA algorithm, as implemented in the ‘linkage’ function of the SciPy [127] (version 1.5.4) library. To reduce structural redundancy for visual representation (Fig. 6), groups of highly similar BGC regions were identified with SciPy’s ‘fcluster’ function (parameters: ‘criterion = “inconsistent”, *t* = 0.1’), and only the medoid within each group was selected for graphical representation (23 out of a total of 42 BGC regions). CoverM (version 0.3.2) was used to measure the average coverage of contigs containing representative siderophore BGCs (parameters: ‘--min-read-percent-identity 0.95 --methods mean’).

### Phylogenetic analysis of nitrogenase genes

To assess the diversity of *nifH* in the *campos rupestres* we fetched all the metagenomic proteins annotated with the K02588 KEGG ortholog and selected orthologs belonging to *nifH* groups I, II, and III [44] from UniProt (release 2020_06) (Supplementary Fig. 5B, labels in gray). Linclust was then used to remove redundancy by clustering identical sequences (parameters: ‘--cluster-mode 2 --cov-mode 1 --min-seq-id 1.0 -c 0.9 --kmer-per-seq 80’). Next, the *nifH* sequences were subjected to a two-step alignment process: they were first aligned with MAFFT (parameters: ‘--auto’) and poorly aligned proteins were identified and removed from the alignment using trimAl [128] (version 1.4.1, parameters: ‘-resoverlap 0.3 -seqoverlap 90’). The remaining sequences were then realigned and unreliable regions were trimmed with trimAl (parameters: ‘-automated1’). Finally, we used IQ-TREE (parameters: ‘-B 1000’) to infer the maximum-likelihood tree from the resulting alignment.

To evaluate the acquisition of the *nif* complex by *Isosphaeraceae* via HGT, we reconstructed a phylogeny using sequences from the *nifH* and *nifD* subunits. In addition to the orthologs encoded by our *Isosphaeraceae* MAGs, we retrieved sequences from all *nif*-encoding *Planctomycetota* (AnnoTree r89), from Frankia, and from selected *Gammaproteobacteria* (UniProt release 2020_06) (Supplementary Fig. 5C). Multiple sequence alignments for *nifH* and *nifD* were obtained using MAFFT (‘--auto’) and trimmed with trimAl (‘-automated1’). The maximum-likelihood *nifHD* tree was inferred with IQ-TREE (‘-B 1000’) using the alignments of both genes in a partitioned model. To further support the HGT hypothesis, we used clinker [129] (version 0.0.16) to compare a *nif*-containing *Isosphaeraceae* contig to the *nif* cluster of *Pseudomonas stutzeri* A1501 (accession: GCA_000013785.1).

### PCR amplification of *nifH*

The *nifH* gene was amplified from total DNA of microbial communities associated with *V. epidendroides* and *B. macrantha* endophytic roots (six samples per species). The reactions were carried out with the primer pair IGK3/DVV [130] using the KAPA2G Robust HotStart ReadyMix kit (KK5705; Basel, Switzerland). The thermocycler program was set to an initial denaturing at 95 °C for 10 min, followed by 34 cycles of denaturing at 95 °C for 15 s, primer annealing at 50 °C for 60 s and extension at 72 °C for 60 s. The products were evaluated in 1.5% agarose gels.

### Identification of prophages and their associated hosts

We used VIBRANT [131] (version 1.2.1) to identify contigs containing prophage sequences. The resulting sequences were then processed by CheckV [132] (version 0.7.0) to remove low-quality fragments and refine the boundaries between host and prophage regions. Auxiliary metabolic genes were identified using KofamScan (KOfam release 2020-05-10). To assign taxonomy to the host, we first removed the predicted viral regions from the prophage-bearing contigs and used MAGpurify2 (version 1.0.0, database available at doi: 10.5281/zenodo.3817702) to classify the host-only segments.

## Supplementary information

Supplementary Note 1

Supplementary Note 2

Supplementary Figure Legends

Supplementary Figure 1

Supplementary Figure 2

Supplementary Figure 3

Supplementary Figure 4

Supplementary Figure 5

Supplementary Tables

## Data Availability

16S rRNA gene and ITS2 region amplicon sequencing data are available at the NCBI Sequence Read Archive (SRA) under the BioProject PRJNA522264. The SRA and IMG identifiers associated with the metagenomic data are listed in Supplementary Table 1. The metagenome-assembled genomes used in this work were deposited in GenBank and their accessions are listed in Supplementary Table 2. Supplementary files containing additional processed data are available at doi: 10.17605/OSF.IO/XKDTV.
