# Supplementary material for: Plant microbiomes harbor potential to promote nutrient turnover in impoverished substrates of a Brazilian biodiversity hotspot
Source: ISME J. 2022 Dec 20;17(3):354–70. doi: 10.1038/s41396-022-01345-1 (PMC9938248; doi:10.1038/s41396-022-01345-1)
Supplement: Supplementary file 3 — Supplementary Figure Legends [file 41396_2022_1345_MOESM3_ESM.docx]

## Supplementary Figure 1

**(A)** Community composition inferred from ITS ASVs at the phylum level. Samples were grouped according to their environment. Bar heights are proportional to the relative abundance of the phylum. Low abundance phyla (relative abundance < 2%) were grouped under the “Other” category. **(B)** Weighted average community identity (WACI) computed from ITS ASV data. Red horizontal lines represent the mean WACI within each sample type. Linear mixed-effects models were used to compare the WACI of below ground environments (substrate and root) and above ground environments (stem and leaves). RX = root (external), RN = root (internal), SX = stem (external), SN = stem (internal), LX = leaf (external), LN = leaf (internal).

## Supplementary Figure 2

**(A)** The Pielou’s equitability index and **(B)** the richness of the studied communities computed from 16S and ITS ASV count data. Red horizontal bars represent the mean diversity within each sample type. **(C, D)** Multidimensional scaling of Bray-Curtis and weighted UniFrac dissimilarities computed from **(C)** 16S and **(D)** ITS ASV data. Samples are colored according to the plant they are associated to, and the shapes represent whether they are from below ground (substrate and root) or above ground (stem and leaves) environments. **(E)** Bar plots representing the fraction of *V. epidendroides*-exclusive, shared, and *B. macrantha*-exclusive ITS ASVs across all sample types. The absolute numbers of ASVs within each group are shown. **(F)** Proportion of the total number of ITS ASVs (light yellow) and of the abundance (dark yellow) that is comprised by ASVs that are shared between the communities associated with both plants. RX = root (external), RN = root (internal), SX = stem (external), SN = stem (internal), LX = leaf (external), LN = leaf (internal).

## Supplementary Figure 3

**(A, B, C)** Phylum-level relative average abundances of genes involved in **(A)** amino acid and organic acid transport, **(B)** carbohydrate degradation, and **(C)** autotrophy in the four studied community types. **(D)** Mean total abundances (in RPKM) of carbohydrate-active enzymes in the substrate and root-associated communities of *V. epidendroides* and *B. macrantha*. **(E)** Mean total abundances (in RPKM) of genes involved in autotrophy in the substrate and root-associated communities. **(F)** Mean total abundances (in fraction of mapped reads) at the family level of MAGs with the metabolic potential for photosynthesis or aerobic carbon monoxide oxidation. The number of MAGs within each family shown within parenthesis. Vertical lines in the bar plots represent the standard error of the mean. RX = root (external), GH = glycoside hydrolases, PL = polysaccharide lyases, CE = carbohydrate esterases, AA = auxiliary activities, PSI = photosystem I, PSII = photosystem II.

## Supplementary Figure 4

**(A)** Phylum-level relative average abundances of genes involved in processes associated with phosphorus mobilization. **(B)** Mean relative abundances of ASVs assigned to the Pseudonocardiaceae family. **(C)** Mean abundance of the *PHO84* transporter across all studied environments. The abundance was measured as the number of *PHO84*-derived reads assigned to the Fungi clade by GraftM. To account for differences in sequencing depth, the total number of reads assigned to this gene in each sample was divided by the sample’s genome equivalents. Vertical lines in the bar plots represent the standard error of the mean. RX = root (external), RN = root (internal), SX = stem (external), SN = stem (internal), LX = leaf (external), LN = leaf (internal).

## Supplementary Figure 5

**(A)** Phylum-level relative average abundances of genes involved in processes associated with nitrogen cycling. **(B)** Maximum-likelihood tree inferred from a dereplicated set of *nifH* orthologs containing reference sequences retrieved from UniProt and proteins predicted from the metagenomes. Branches are colored according to the major *nifH* group they belong to. The tree was rooted at the lowest common ancestor node of the group III *nifH*. The *Rhizobiales* and *Isosphaeraceae* clades are indicated by the colored backgrounds. Reference sequences from UniProt are indicated by gray labels and MAG-encoded orthologs are shown with bold labels. Not all MAG-encoded orthologs were shown because some clustered together. The remaining tips are labelled using the most specific rank of the taxonomic lineage assigned to the *nifH*-containing contig (full lineages are show at the below the tree). **(C)** Maximum-likelihood tree inferred from *nifH* and *nifD* orthologs from the *Isosphaeraceae* MAGs, selected *Gammaproteobacteria* with high sequence identity to MAG-encoded proteins, *Frankia alni*, and other *nif*-encoding *Planctomycetota*. The two major nif groups are indicated by gray labels over the branches leading to their respective lowest common ancestor nodes. The tree was rooted in the node between the two groups. The numbers associated with internal branches represent the percentage of bootstraps that support it. Bootstrap values lower than 70% were omitted. **(D)** Synteny between a *Isosphaeraceae* contig containing the *nif* cluster and a region of the *Pseudomonas stutzeri* genome. Arrow heads below the *Isosphaeraceae* contig indicate whether the genes were assigned to *Planctomycetota* (pink) or *Gammaproteobacteria* (green). Connections between genes with pairwise identities below 40% are omitted. **(E)** Mean relative abundances of ASVs assigned to the *Bradyrhizobium* genus. **(F)** Gel image of PCR products for *nifH* from total DNA of microbial communities associated with the endophytic root of *V. epidendroides* and *B. macrantha*. The average expected fragment size for positive PCRs is 382 bp. The gel image was inverted from black to white. Leftmost and rightmost columns: Ladder, R1–6: biological replicate 1 to 6, C1 = Positive control 1 (*Azospirillum brasilense* Abv5), C2 = Positive control 2 (*Bradyrhizobium japonicum* SEMIA 5079), C3 = Positive control 3 (*Bradyrhizobium diazoefficiens* SEMIA 5080), E = *Escherichia coli*, N = Negative control. **(G)** Mean abundance of the *Bradyrhizobium* *nifH* transporter across all studied environments. The abundance was measured as the number of *nifH*-derived reads assigned to the *Bradyrhizobium* clade by GraftM. To account for differences in sequencing depth, the total number of reads assigned to this gene in each sample was divided by the sample’s genome equivalents. **(H)** *Bradyrhizobium* contig carrying a provirus containing the *exoZ* gene (in red). **(I)** Mean total abundances (in fraction of mapped reads) at the family level of MAGs with the metabolic potential for nitrogen fixation, ammonia oxidation, or hydroxylamine oxidation. The number of MAGs within each family shown within parenthesis. Vertical lines in the bar plots represent the standard error of the mean. RX = root (external), RN = root (internal), SX = stem (external), SN = stem (internal), LX = leaf (external), LN = leaf (internal).
