# Supplementary figures and images for: Plant microbiomes harbor potential to promote nutrient turnover in impoverished substrates of a Brazilian biodiversity hotspot
Source: ISME J. 2022 Dec 20;17(3):354–70. doi: 10.1038/s41396-022-01345-1 (PMC9938248; doi:10.1038/s41396-022-01345-1)

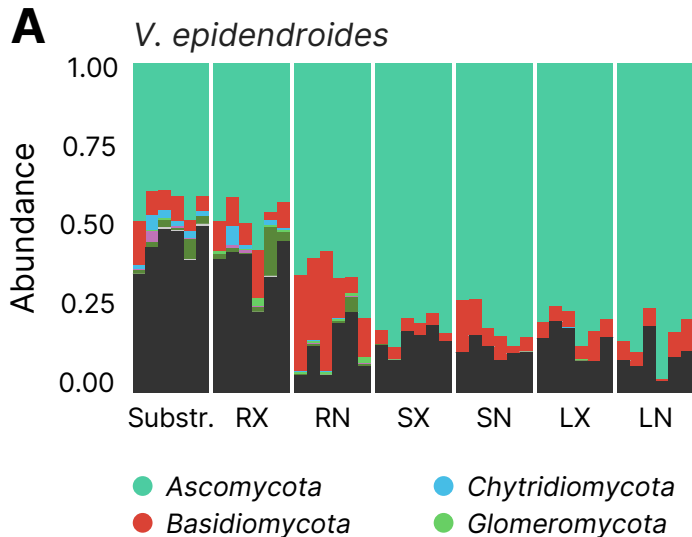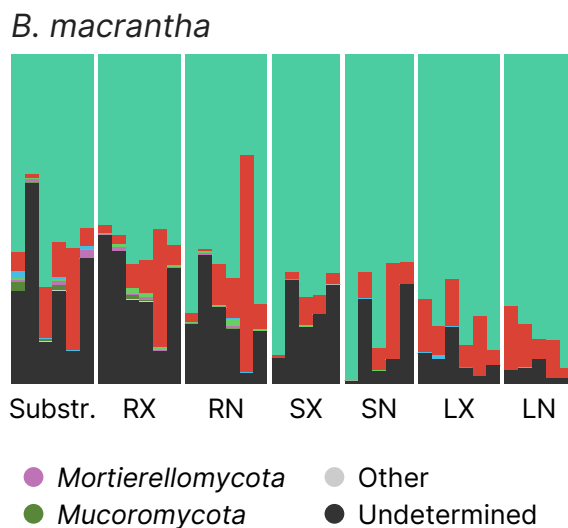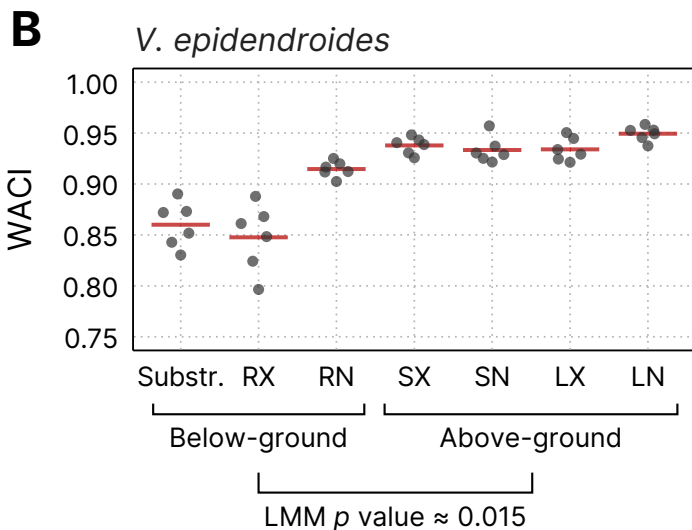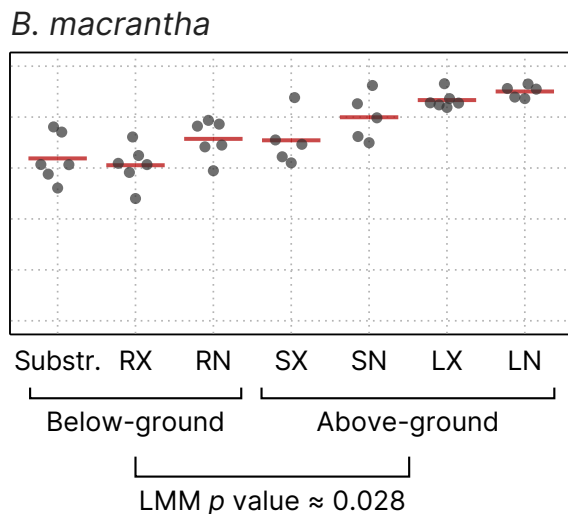

Supplement: Supplementary file 4 — Supplementary Figure 1 [file 41396_2022_1345_MOESM4_ESM.pdf]

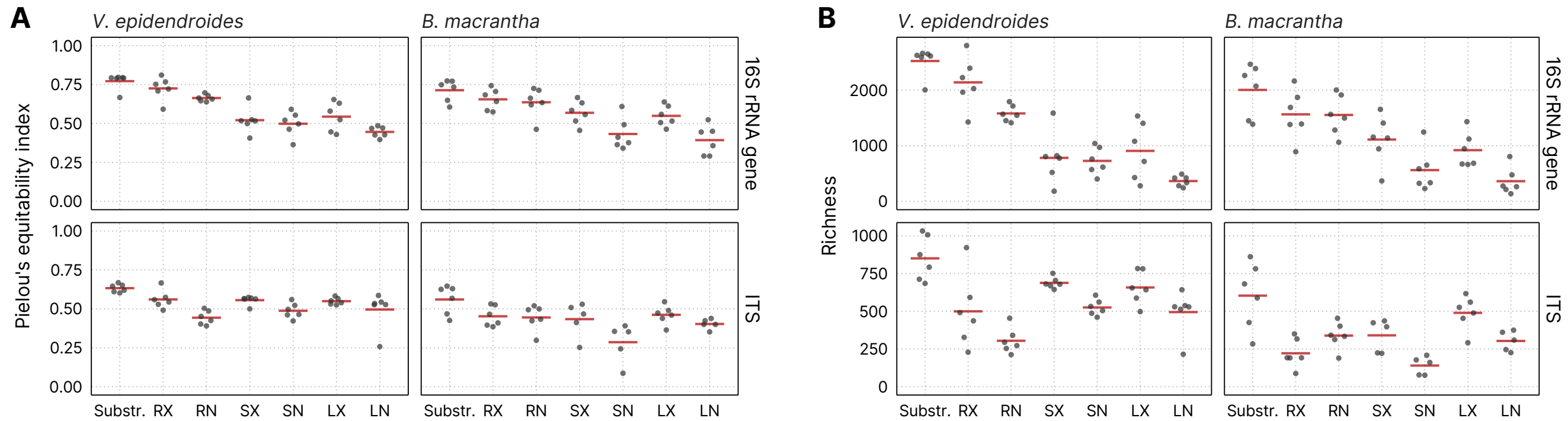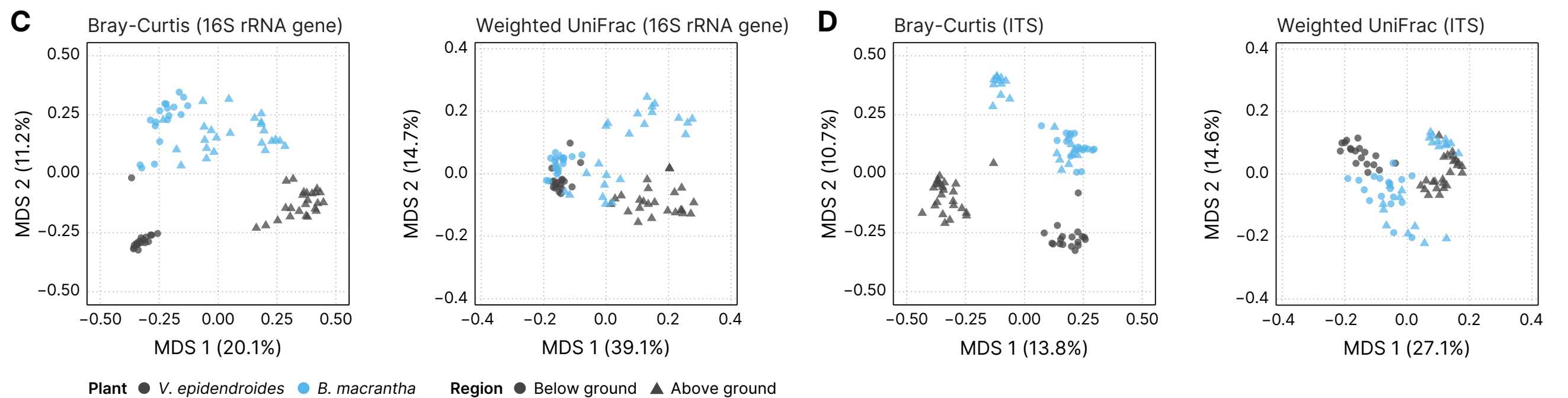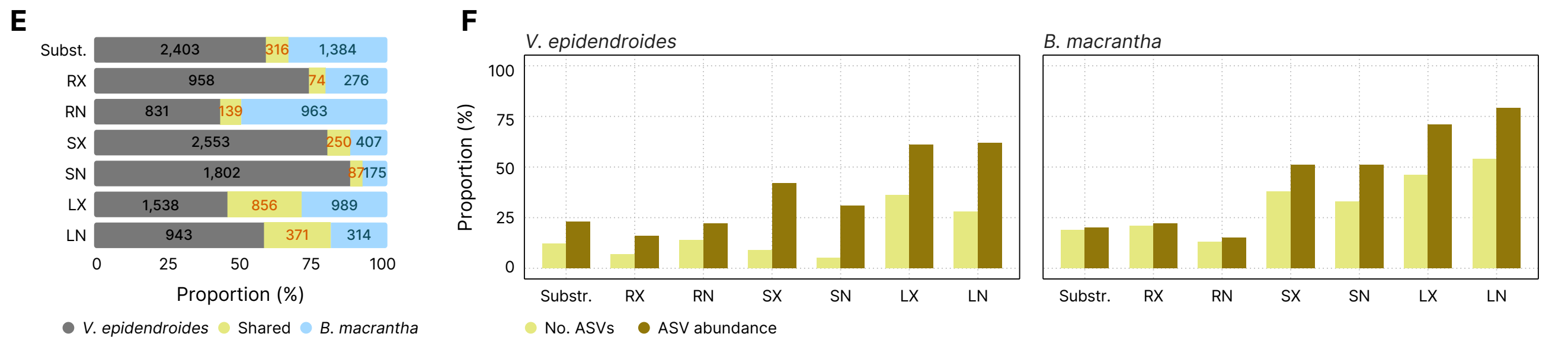

Supplement: Supplementary file 5 — Supplementary Figure 2 [file 41396_2022_1345_MOESM5_ESM.pdf]

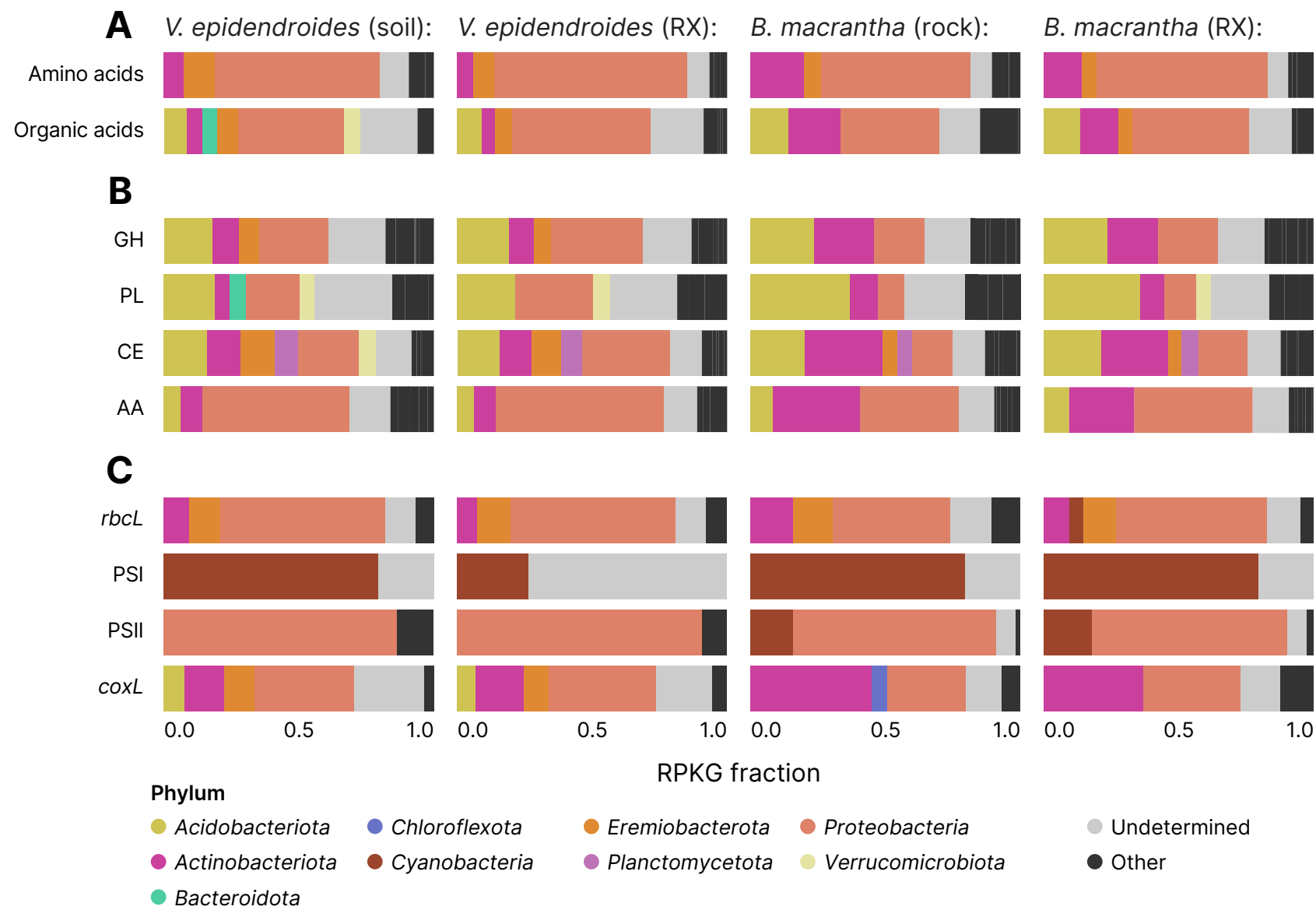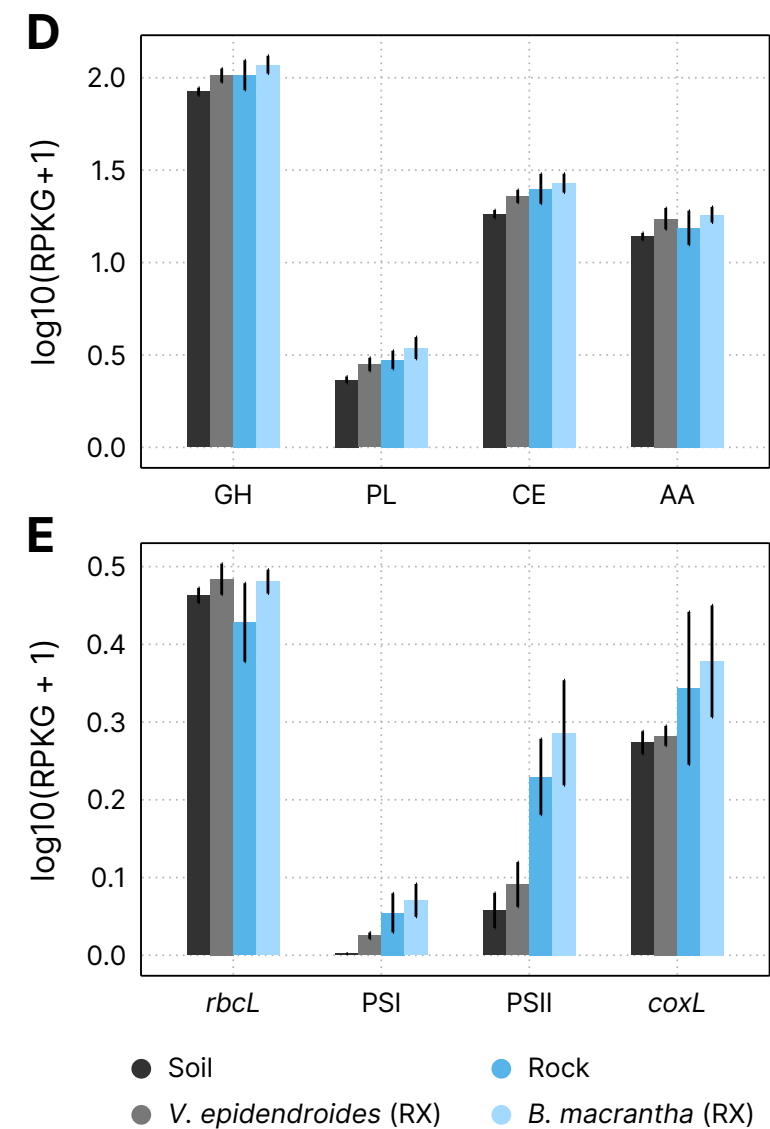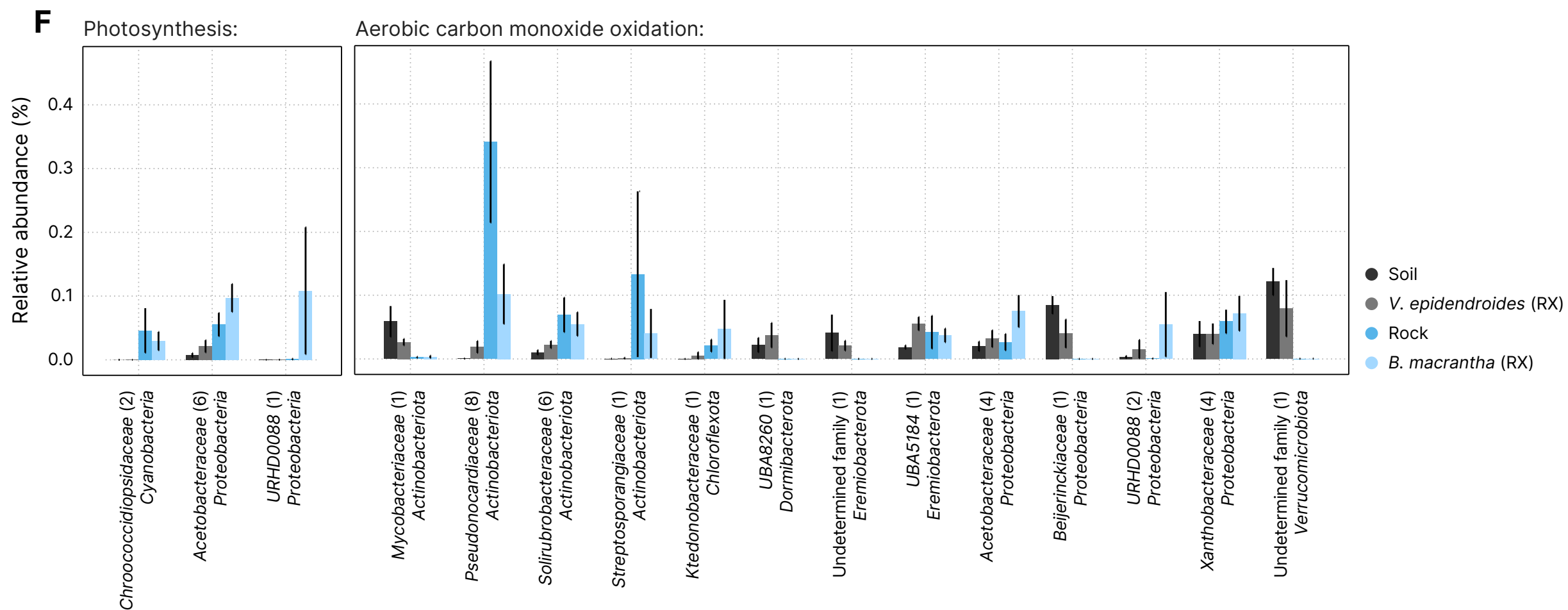

Supplement: Supplementary file 6 — Supplementary Figure 3 [file 41396_2022_1345_MOESM6_ESM.pdf]

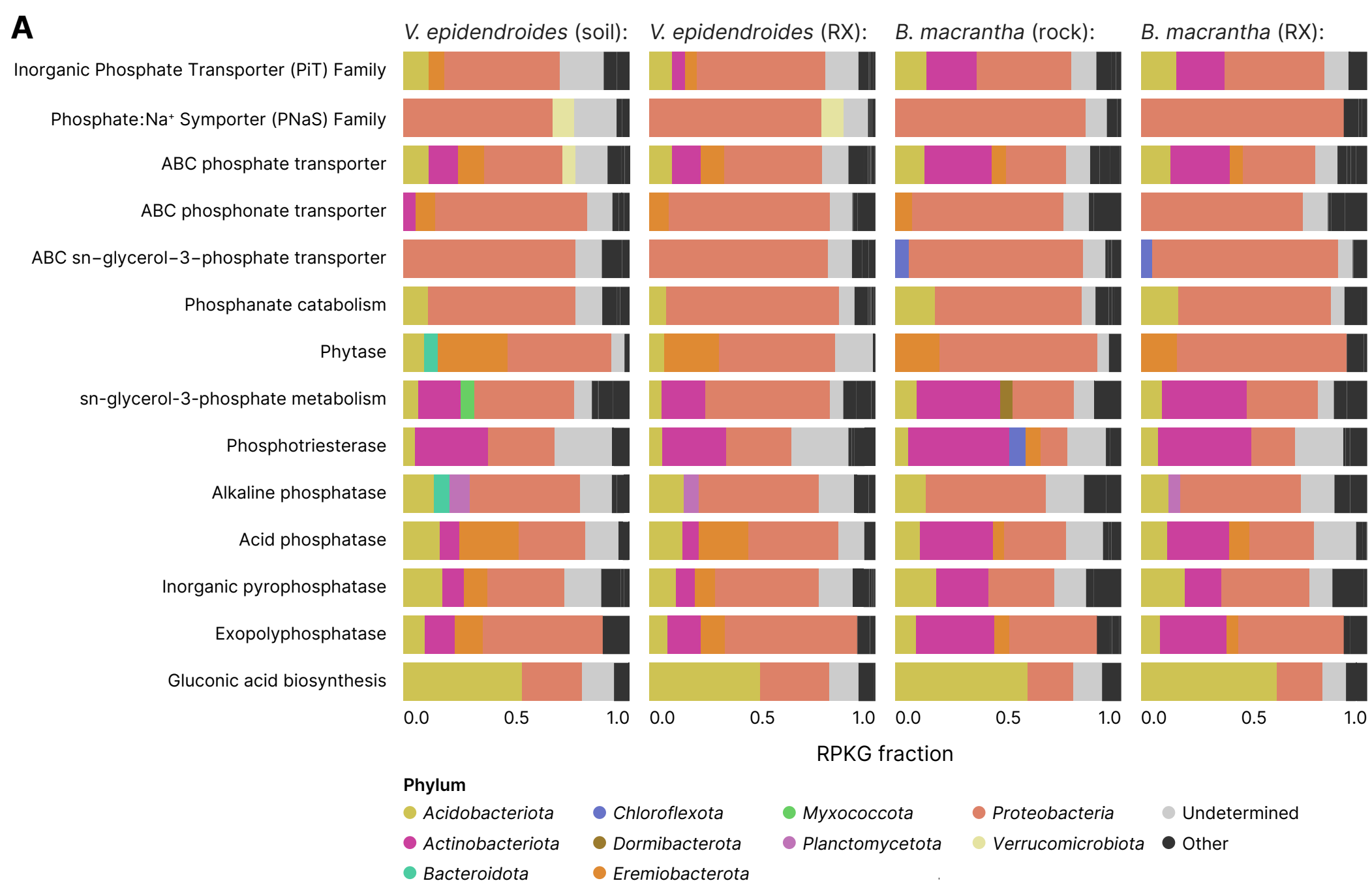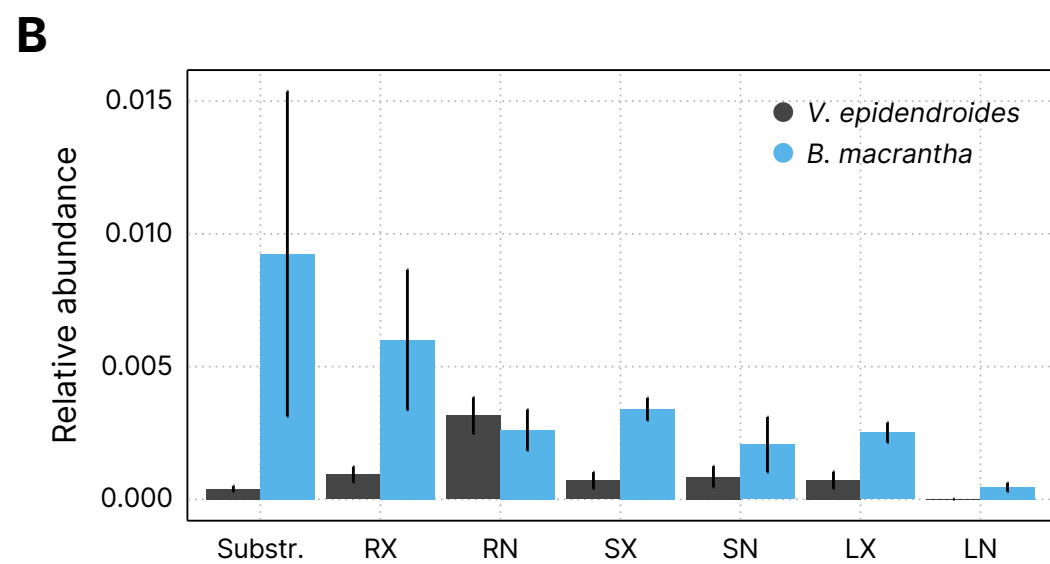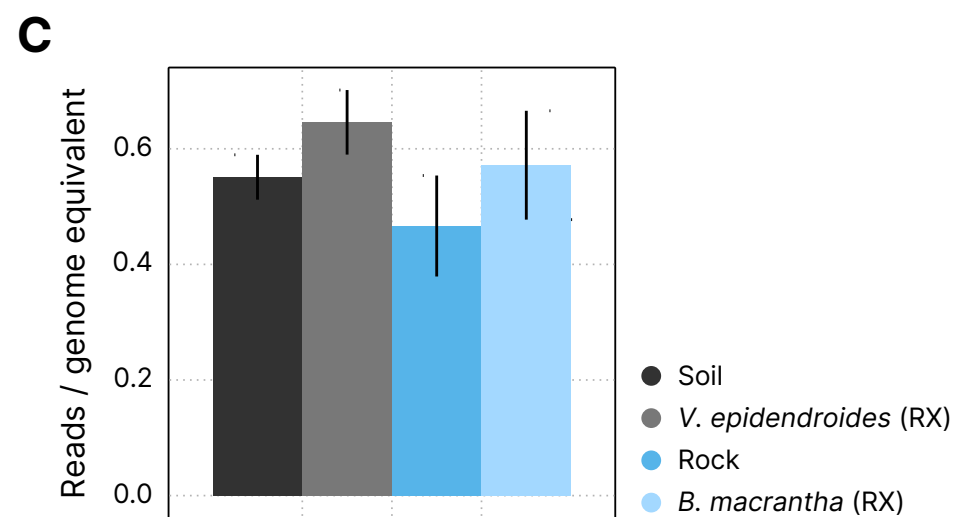

Supplement: Supplementary file 7 — Supplementary Figure 4 [file 41396_2022_1345_MOESM7_ESM.pdf]
