# Supplementary material for: Plant microbiomes harbor potential to promote nutrient turnover in impoverished substrates of a Brazilian biodiversity hotspot
Source: ISME J. 2022 Dec 20;17(3):354–70. doi: 10.1038/s41396-022-01345-1 (PMC9938248; doi:10.1038/s41396-022-01345-1)
Supplement: Supplementary file 8 — Supplementary Figure 5 [file 41396_2022_1345_MOESM8_ESM.pdf]

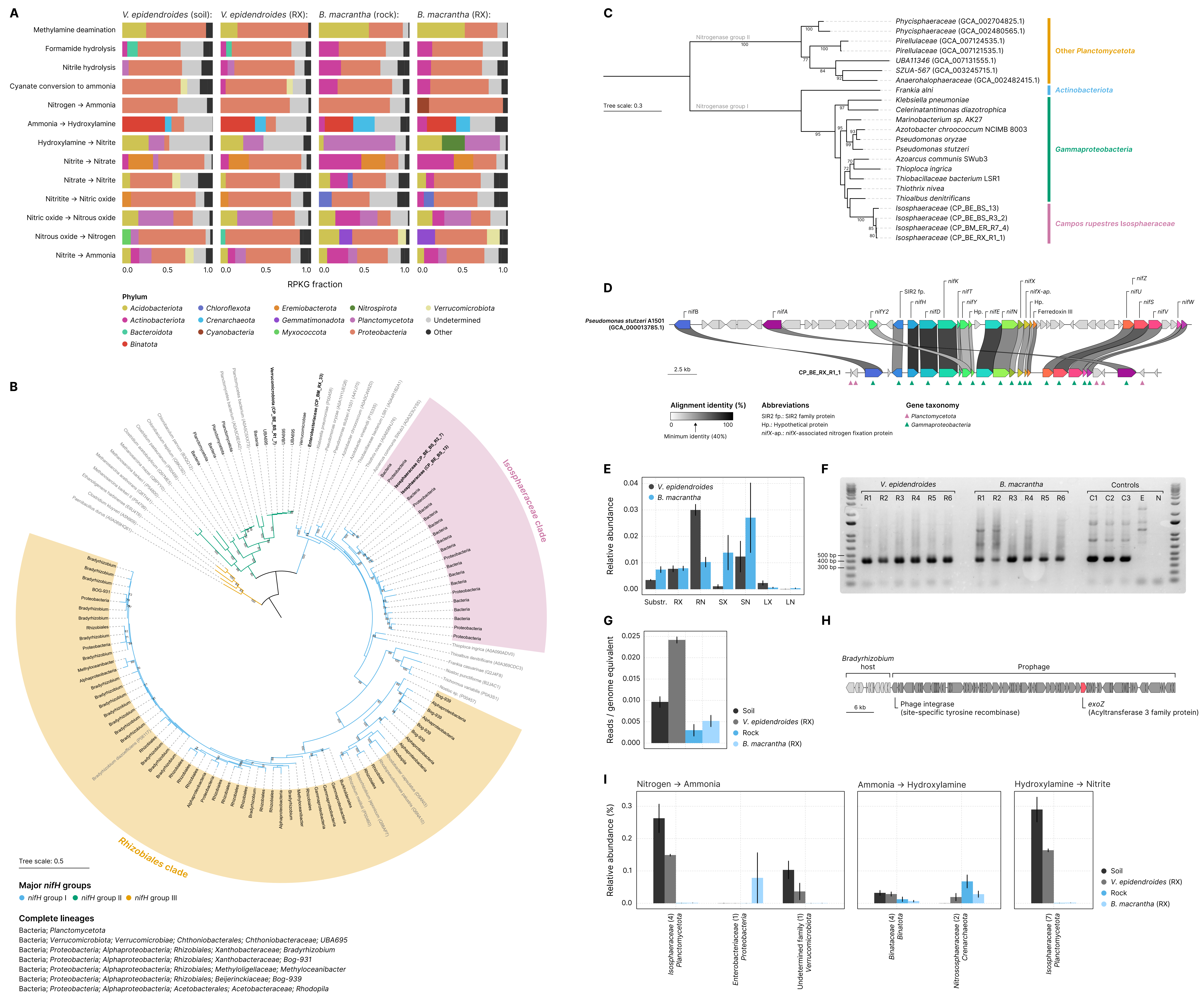
A
V. epidendroides (soil):
V. epidendroides (RX):
B. macrantha (rock):
B. macrantha (RX):
Methylamine deamination
Formamide hydrolysis
Nitrile hydrolysis
Cyanate conversion to ammonia
Nitrogen → Ammonia
Ammonia → Hydroxylamine
Hydroxylamine → Nitrite
Nitrite → Nitrate
Nitrate → Nitrite
Nitritite → Nitric oxide
Nitric oxide → Nitrous oxide
Nitrous oxide → Nitrogen
Nitrite → Ammonia
0.0
0.5
1.0
RPKG fraction
Phylum
Acidobacteriota
Actinobacteriota
Bacteroidota
Binatota
Chloroflexota
Crenarchaeota
Cyanobacteria
Eremiobacterota
Gemmatimonadota
Myxococcota
Nitrospirota
Planctomycetota
Proteobacteria
Verrucomicrobiota
Undetermined
Other
B
Isosphaeraceae clade
Rhizobiales clade
Tree scale: 0.5
Major nifH groups
nifH group I
nifH group II
nifH group III
Complete lineages
Bacteria; Planctomycetota
Bacteria; Verrucomicrobiota; Verrucomicrobiae; Chthoniobacterales; Chthoniobacteraceae; UBA695
Bacteria; Proteobacteria; Alphaproteobacteria; Rhizobiales; Xanthobacteraceae; Bradyrhizobium
Bacteria; Proteobacteria; Alphaproteobacteria; Rhizobiales; Xanthobacteraceae; Bog-931
Bacteria; Proteobacteria; Alphaproteobacteria; Rhizobiales; Methyloligellaceae; Methyloceanibacter
Bacteria; Proteobacteria; Alphaproteobacteria; Rhizobiales; Beijerinckiaceae; Bog-939
Bacteria; Proteobacteria; Alphaproteobacteria; Acetobacterales; Acetobacteraceae; Rhodopila
C
Nitrogenase group II
Nitrogenase group I
Tree scale: 0.3
Phycisphaeraceae (GCA_002704825.1)
Phycisphaeraceae (GCA_002480565.1)
Pirellulaceae (GCA_007124535.1)
Pirellulaceae (GCA_007121535.1)
UBA11346 (GCA_007131555.1)
SZUA-567 (GCA_003245715.1)
Anaerohalophaeraceae (GCA_002482415.1)
Frankia alni
Klebsiella pneumoniae
Celerinatantimonas diazotrophica
Marinobacterium sp. AK27
Azotobacter chroococcum NCIMB 8003
Pseudomonas oryzae
Pseudomonas stutzeri
Azoarcus communis SWub3
Thioploca ingrica
Thiobacillaceae bacterium LSR1
Thiothrix nivea
Thioalbus denitrificans
Isosphaeraceae (CP_BE_BS_13)
Isosphaeraceae (CP_BE_BS_R3_2)
Isosphaeraceae (CP_BM_ER_R7_4)
Isosphaeraceae (CP_BE_RX_R1_1)
Other Planctomycetota
Actinobacteriota
Gammaproteobacteria
Campos rupestres Isosphaeraceae
D
Pseudomonas stutzeri A1501 (GCA_000013785.1)
CP_BE_RX_R1_1
2.5 kb
nifB
nifA
nifY2
SIR2 fp.
nifH
nifD
nifK
nifT
nifY
Hp.
nifE
nifN
nifX
nifX-ap.
Ferredoxin III
nifU
nifS
nifV
nifZ
nifW
Alignment identity (%)
0
100
Minimum identity (40%)
Abbreviations
SIR2 fp.: SIR2 family protein
Hp.: Hypothetical protein
nifX-ap.: nifX-associated nitrogen fixation protein
Gene taxonomy
Planctomycetota
Gammaproteobacteria
E
V. epidendroides
B. macrantha
Relative abundance
Substr.
RX
RN
SX
SN
LX
LN
F
V. epidendroides
B. macrantha
Controls
R1 R2 R3 R4 R5 R6
C1 C2 C3 E N
500 bp
400 bp
300 bp
G
Reads / genome equivalent
Soil
V. epidendroides (RX)
Rock
B. macrantha (RX)
H
Bradyrhizobium host
Prophage
6 kb
Phage integrase (site-specific tyrosine recombinase)
exoZ (Acyltransferase 3 family protein)
I
Nitrogen → Ammonia
Ammonia → Hydroxylamine
Hydroxylamine → Nitrite
Relative abundance (%)
Isosphaeraceae (4) Planctomycetota
Enterobacteriaceae (1) Proteobacteria
Undetermined family (1) Verrucomicrobiota
Binataceae (4) Binatota
Nitrososphaeraceae (2) Crenarchaeota
Isosphaeraceae (7) Planctomycetota
Soil
V. epidendroides (RX)
Rock
B. macrantha (RX)
